# Synthesis and physical properties of tris(dialkylamino)cyclopropenium dicyanamide ionic liquids†

**DOI:** 10.1039/c8ra05558k

**Published:** 2018-08-07

**Authors:** Owen J. Curnow, Matthew I. J. Polson, Kelvin J. Walst, Ruhamah Yunis

**Affiliations:** School of Physical and Chemical Sciences, University of Canterbury Private Bag 4800 Christchurch 8041 New Zealand owen.curnow@canterbury.ac.nz +64 3 369 4239

## Abstract

The synthesis and properties of 16 tris(dialkylamino)cyclopropenium (TDAC) cations with the dicyanamide (DCA) anion, [N(CN)_2_]^−^, are described. *D*_3h_- and *C*_3h_-symmetric cations ([C_3_(NR_2_)_3_]DCA (R = Me, Et, Pr, Bu, Pent, Hex, Dec) and [C_3_(NRMe)_3_]DCA (R = Bu, St), respectively) were synthesised by reaction of C_3_Cl_5_H with the corresponding amine. Reaction of the alkoxydiaminocyclopropenium salt [C_3_(NEt_2_)_2_(OMe)]^+^ with amines led to a series of *C*_2v_-symmetric salts [C_3_(NEt_2_)_2_(NR_2_)]DCA (R = Me, Bu, Hex) and two *C*_s_-symmetric salts and [C_3_(NEt_2_)_2_(NRMe)]DCA (R = Me, Bu). Similarly, [C_3_(NMe_2_)_2_(OMe)]^+^, was used to prepare the *C*_s_-symmetric salts [C_3_(NMe_2_)_2_(NRMe)]DCA (R = Pr, Bu). In addition to characterisation by NMR, mass spectrometry and microanalysis, the salts were characterised by DSC, TGA, density, viscosity, conductivity and miscibility/solubility studies. Comparisons have been made with similar series of bistriflimide (NTf_2_^−^) salts that have been previously reported to see whether the same trends are observed with a different anion.

## Abstract 2

An investigation of symmetry (*D*_3h_, *C*_3h_, *C*_2v_ and *C*_s_) and alkyl chain length (6–60 alkyl carbon atoms) effects on the physical properties of peralkylated triaminocyclopropenium dicyanamide salts.

## Graphical abstract

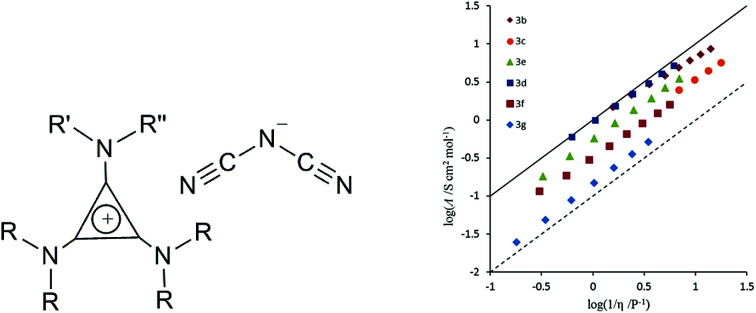

## 1. Introduction

Ionic liquids (ILs) are an increasingly important class of soft materials that have undergone rapid development in recent years; many of these materials are now commercially available.^1^ These materials are frequently considered as green alternatives to classical organic solvents due to their general properties of almost zero vapour pressure, low flammability, tunability, excellent solvent properties and potential for efficient recycling.^2^ Additionally, their conductivity and other useful properties have led to many applications.^3^

Traditionally, there are four major cation-based classes of ILs: imidazolium, pyridinium, phosphonium and ammonium. Although cyclopropenium salts have been known since the first report of [C_3_Ph_3_]^+^ in 1957 (ref. 4) and the triaminocyclopropenium (TAC) salts have been known for more than 40 years,^5^ we did not report on the IL properties of TAC salts until 2011.^6^ TAC salts are remarkably stable due to a combination of the aromaticity of the 2π-electron ring system as well as the strong π donation from the three amino groups. TAC salts more generally have been recently reviewed by Komatsu and Kitagawa as well as Bandar and Lambert.^7,8^ TAC cations have greater charge delocalisation than ammonium, phosphonium, and guanidinium cations, while they have reduced hydrogen bond donor capabilities, compared to the imidazolium, pyridinium and triazonium cations, due to a lack of aromatic C–H groups. The high-lying non-bonding HOMO of TAC cations gives particularly weak cation–anion interactions^9^ and their salts consequently have unusual properties: in some early work, Weiss and co-workers prepared iodide–iodoacetylene and iodide–iodoarene adducts.^10^ More recently, we isolated an interesting discrete dichloride hexahydrate cube, [Cl_2_(H_2_O)_6_]^2−^, in which the solid state structure is very similar to the calculated structure.^11^ Even more surprising is that, upon removal of the solvate, the cations form dicationic pairs with remarkably short π–π stacking distances.^12^ In 2015, we reported on a series of tris(dialkylamino)cyclopropenium (TDAC) NTf_2_^−^ ILs in which the effects of cation size and symmetry were investigated. Cation symmetry classes included *D*_3h_, *C*_3h_, *C*_2v_ and *C*_s_ while the cation size varied from the hexamethyl cation [C_3_(NMe_2_)_3_]^+^ to the hexadecyl cation [C_3_(N(C_10_H_21_)_2_)_3_]^+^.^13^ Variation of the alkyl chain length in IL cations is generally used to fine tune the properties of an IL. In contrast, exchanging the anion frequently leads to much larger step changes in properties such as the viscosity, conductivity and solubility/miscibility profiles. However, the properties of an ionic liquid are not just the sum of the cations and the anions, the properties are also dependent on how the anions and cations interact: for example, their relative sizes, shapes and charge distributions, as well as any hydrogen bonding, impact on the intermolecular forces and thus their physical properties. Therefore, we sought to investigate how exchanging from one anion to another impacts on trends in the physical properties of TAC ILs. Following our detailed study on bistriflimide salts, we now report on the corresponding dicyanamide ([N(CN)_2_]^−^, DCA) TDAC salts, an anion which would be expected to produce lower viscosities, higher conductivities and greater hydrophilicity. Some of this work has been communicated.^6,14^

## 2. Results and discussion

### 2.1 Synthesis

**Scheme 1:**
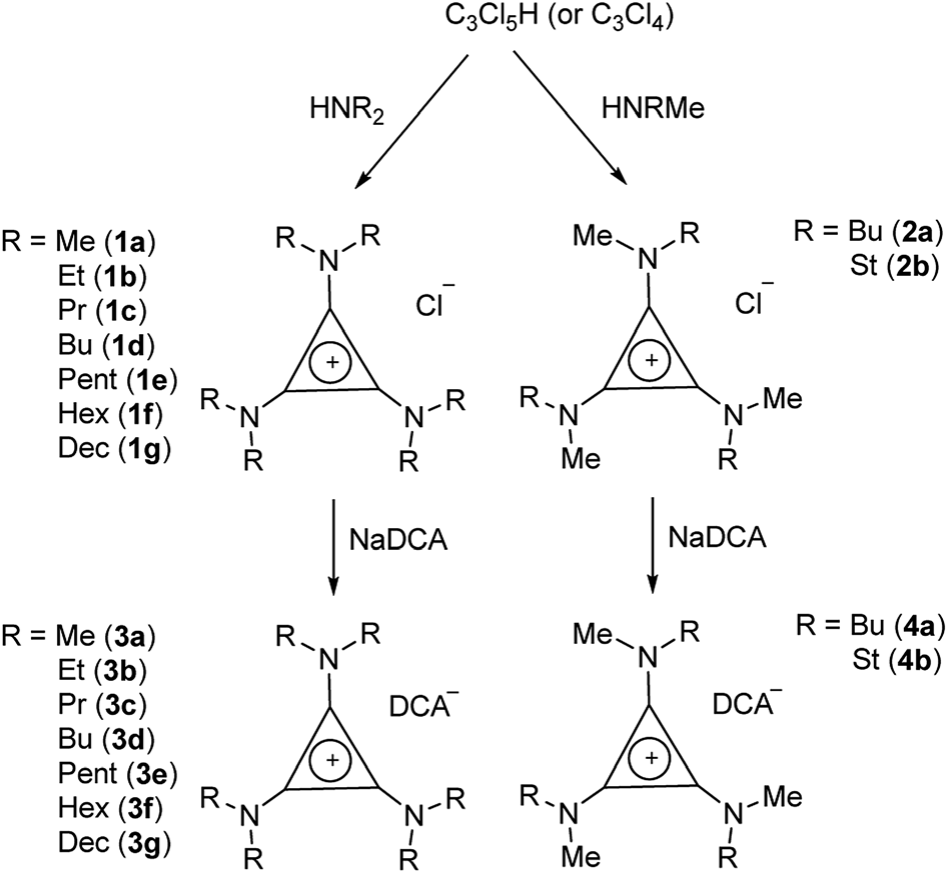
Synthesis of *D*_3h_- and *C*_3h_-symmetric TDAC salts.

TDAC salts were first prepared by reaction of tetrachlorocyclopropene with secondary amines,^5^ and more recently by reaction with pentachlorocyclopropane.^6,14,15^ These routes (Scheme 1) provide the *D*_3h_- and *C*_3h_-symmetric cation chloride salts [C_3_(NR_2_)_3_]Cl (1) and [C_3_(NRR′)_3_]Cl (2), respectively. Treatment of these salts with aqueous NaDCA readily provides the corresponding dicyanamide salts (3 and 4, respectively) which can be extracted into an organic solvent such as chloroform or dichloromethane. Earlier, we communicated the syntheses and some properties of the [C_3_(NR_2_)_3_]DCA salts for R = Et (3b)^14^ and Bu (3d).^6^ Here we additionally include the related syntheses for R = Me (3a), Pr (3c), Pent (3e), Hex (3f) and Dec (3g) as well as [C_3_(NBuMe)_3_]DCA (4a) and [C_3_(NStMe)_3_]DCA (4b, St = C_18_H_37_).

**Scheme 2:**
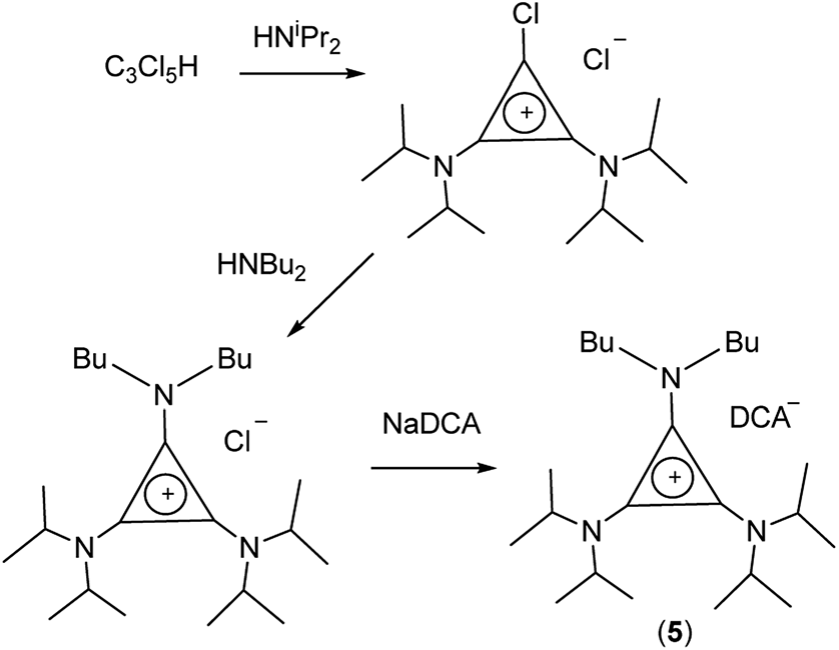
Synthesis of [C_3_(N^i^Pr_2_)_2_(NBu_2_)]DCA (5).

**Scheme 3:**
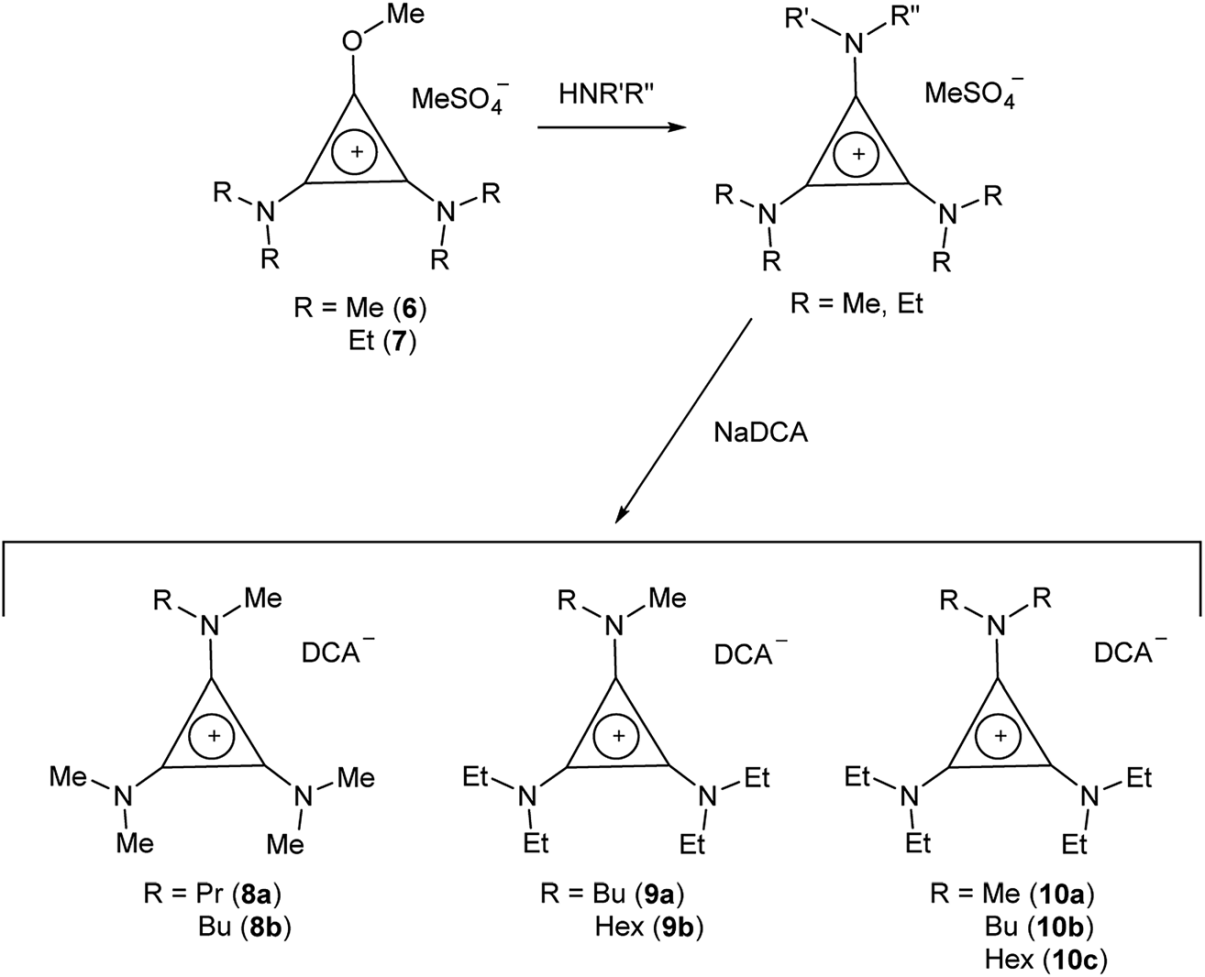
Synthesis of *C*_2v_- and *C*_s_-symmetric TDAC salts.

If the secondary amine is bulky, such as HN^i^Pr_2_ or HN(C_6_H_11_)_2_, then its reaction with C_3_Cl_5_H or C_3_Cl_4_ gives the corresponding diaminochlorocyclopropenium cation [C_3_(NR_2_)_2_Cl]^+^ which can then be treated with a smaller secondary amine to provide a limited range of cations with *C*_2v_ and *C*_s_ symmetry: [C_3_(NR_2_)_2_(NR′_2_)]^+^ and [C_3_(NR_2_)_2_(NR′R′′)]^+^, respectively, in which R is bulky and NR′R′′ is reasonably small.^16,17^ Here we have used this route (Scheme 2) to prepare [C_3_(N^i^Pr_2_)_2_(NBu_2_)]DCA (5). Due to the limited versatility of this route, we developed a route *via* reaction of secondary amines with the alkoxydiaminocyclopropenium cations [C_3_(NMe_2_)_2_(OMe)]^+^ (6) and [C_3_(NEt_2_)_2_(OMe)]^+^ (7) (Scheme 3). As with the chloride salts, addition of aqueous NaDCA followed by extraction with an organic solvent allows one to isolate the DCA salts. Scheme 3 illustrates the three series of ILs that were prepared in this way: two *C*_s_-symmetric series, 8 and 9, *via*6 and 7, respectively; and a *C*_2v_-symmetric series, 10, prepared *via*7. Earlier, we communicated the syntheses and some properties of the salts [C_3_(NEt_2_)_2_(NRMe)]DCA (R = Bu (9a), Hex (9b)) and [C_3_(NEt_2_)_2_(NR_2_)]DCA (R = Me (10a), Bu (10b), Hex (10c)).^14^ When looking at trends within these series, note that some higher-symmetry species may also belong to these series, *i.e.*, 3a can be included in series 8; 3b in series 10; and 10a in series 9. Similarly, 3a can be considered part of the *C*_3h_4 series of cations.

**Scheme 4:**
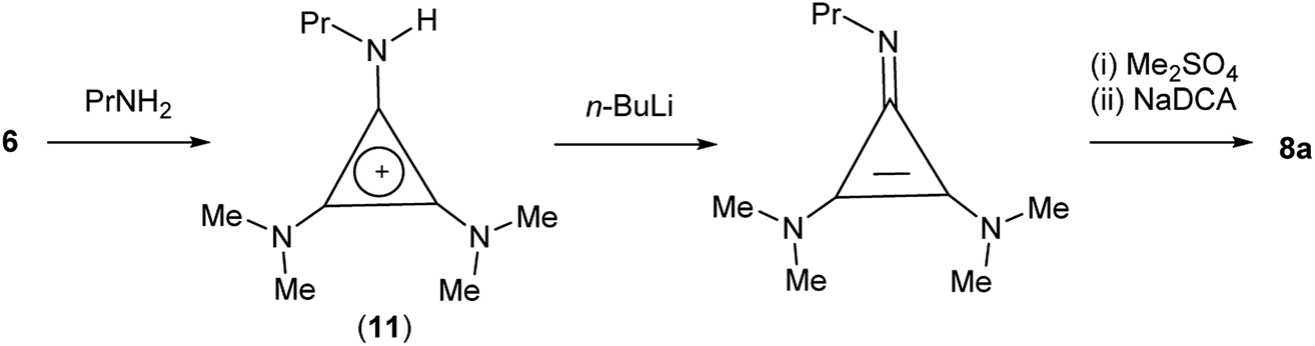
Alternative synthesis of *C*_s_-symmetric 8a.

TDAC cations can also be prepared by alkylation of a cyclopropenimine after deprotonation of the corresponding protic TAC cation. In the case of 8a, it was more convenient (and cheaper) to use this route *via* the protic TAC cation [C_3_(NMe_2_)_2_(NPrH)]^+^ (11) (Scheme 4).

All new compounds were characterised by ^1^H- and ^13^C{^1^H}-NMR spectroscopy as well as ES-MS and microanalysis. Chloride and water contents were determined for ILs prior to measurement of their physical properties; namely, DSC, TGA, viscosity, conductivity and density. These impurities in particular are known to impact on the physical properties of ILs.

With respect to the NMR spectra, it should firstly be borne in mind that rotation about the exocyclic C–N bonds is fast on the NMR timescale; thus, a *C*_s_-symmetric cation such as 8 exhibits *C*_2v_ symmetry on the NMR timescale, and there is only one ^1^H and ^13^C-NMR signal for the NMe_2_ groups. Typical ^1^H- and ^13^C-NMR ranges were tabulated previously.^13^

### 2.2 DSC data

DSC data were collected at 10 °C min^−1^ and the results are given in Table 1. Not surprisingly, the only salt that is not an IL by definition (arbitrarily defined as a melting point less than 100 °C) is that with the smallest and most symmetric (*D*_3h_) cation, 3a, with a melting point (*T*_m_) of 117 °C. A large number of factors are known to influence *T*_m_: the various intermolecular forces (coulombic, van der Waals, hydrogen-bonding *etc.*), conformational flexibility and the shape or symmetry of the species. Generally, it is found that ILs have high melting points for small ions in which coulombic attractions dominate. They also have high melting points for large ions in which van der Waals interactions dominate. Reducing the symmetry or increasing the conformational flexibility of the side chains tends to reduce *T*_m_ since they reduce the factors that favour efficient packing in the solid state.

**Table 1:** DSC (10 °C min^−1^) and TGA data for DCA salts

Cation	*T*_g_/°C	*T*_m_/°C	*T*_d_/°C, 1 °C min^−1^	*T*_d_/°C, 10 °C min^−1^
[C_3_(NMe_2_)_3_]^+^ (3a)	—	117	255	285
[C_3_(NEt_2_)_3_]^+^ (3b)	—	9	291	330
[C_3_(NPr_2_)_3_]^+^ (3c)	—	53	295	317
[C_3_(NBu_2_)_3_]^+^ (3d)	−62	17	293	335
[C_3_(NPent_2_)_3_]^+^ (3e)	−64	—	292	321
[C_3_(NHex_2_)_3_]^+^ (3f)	−64	—	291	321
[C_3_(NDec_2_)_3_]^+^ (3g)	—	−13	276	321
[C_3_(NBuMe)_3_]^+^ (4a)	−73	—	298	331
[C_3_(NStMe)_3_]^+^ (4b)	—	52	241	279
[C_3_(N(i-Pr)_2_)_2_NBu_2_]^+^ (5)	−52	47	302	332
[C_3_(NMe_2_)_2_NPrMe]^+^ (8a)	−74	—	233	257
[C_3_(NMe_2_)_2_NBuMe]^+^ (8b)	−73	—	259	287
[C_3_(NEt_2_)_2_NBuMe]^+^ (9a)	−83	8	287	332
[C_3_(NEt_2_)_2_NHexMe]^+^ (9b)	−82	—	301	342
[C_3_(NEt_2_)_2_NMe_2_]^+^ (10a)	−85	32	293	322
[C_3_(NEt_2_)_2_NBu_2_]^+^ (10b)	−82	30	302	334
[C_3_(NEt_2_)_2_NHex_2_]^+^ (10c)	−80	—	304	348

**Fig. 1:**
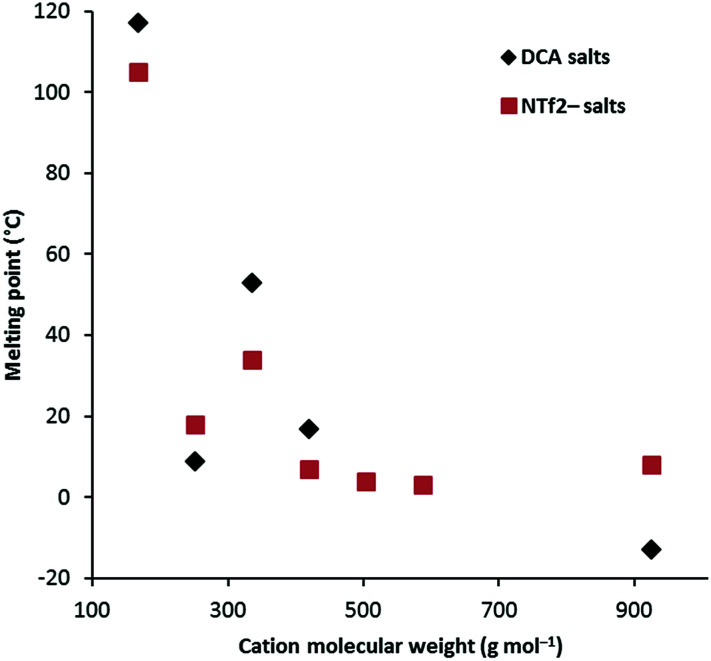
Melting points of the *D*_3h_-symmetry classes (salts 3).

Fig. 1 shows a plot of *T*_m_*versus* cation molecular weight (MW) for the *D*_3h_ class of cations for both the NTf_2_^−^ salts as well as the DCA salts. This shows a rapid drop in *T*_m_ from 3a as both size and conformational flexibility rapidly increase. Generally the DCA salts might be expected to have higher *T*_m_ due to closer electrostatic attractions to the cation, however, the hexaethyl and hexadecyl salts have lower *T*_m_ and, more generally, the TDAC NTf_2_^−^ salts do not show a clear trend towards lower *T*_m_; presumably the greater size difference in the hexadecyl case explains that one. Unfortunately, *T*_m_ transitions were not identified for the hexapentyl and hexahexyl salts. Remarkably, the hexapropyl salts have a higher *T*_m_ than the hexaethyl and hexabutyl analogues for both the NTf_2_^−^ and DCA salts. This may be a result of chain flexibility issues. There is insufficient data to discern clear trends for the other series. Notably, 4b, with very long C_18_ chains, has a significantly higher *T*_m_ than the similarly-sized hexadecyl salt 3g. That is also the case for the NTf_2_^−^ analogues. Salt 5 also has a significantly higher *T*_m_ (47 °C) than similarly-sized salts, and this can be attributed to the lack of flexibility of the isopropyl groups.

### 2.3 Thermal decomposition

**Fig. 2:**
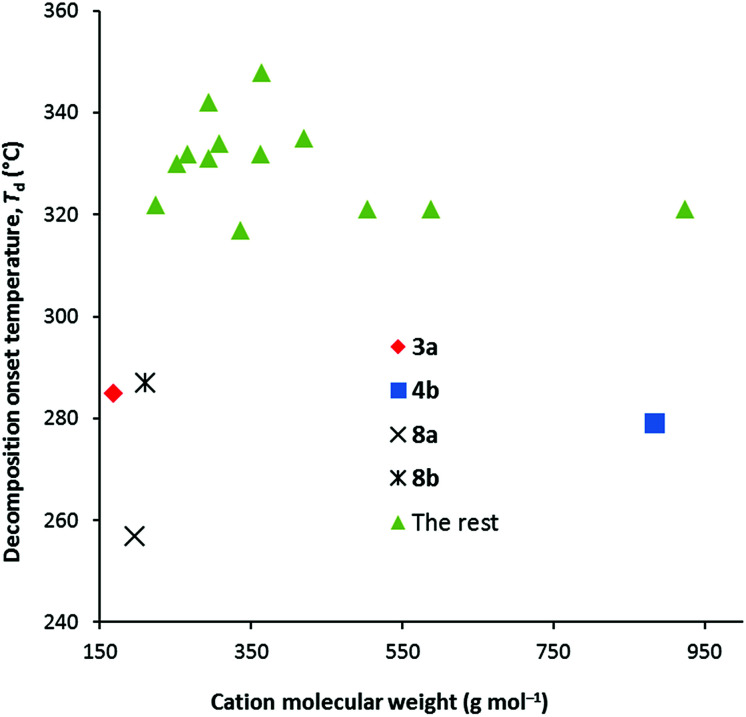
*T*_d_ values of the TDAC DCA salts at 10 °C min^−1^.

Thermal decomposition data were collected at both 1 °C min^−1^ and 10 °C min^−1^ and are given in Table 1. We found with the NTf_2_^−^ salts that the major factor in determining the thermal decomposition onset temperature, *T*_d_, is the number of methyl groups, and especially the number of dimethylamino groups. When none of the six alkyl groups are methyl groups, *T*_d_ is relatively invariant: at 1 °C min^−1^, *T*_d_ ranges 275–305 °C for the DCA salts *versus* 343–364 °C for NTf_2_^−^ salts, while at 10 °C min^−1^, it ranges from 315–350 °C for the DCA salts *versus* 393–409 °C for the NTf_2_^−^ salts. Typically then, the DCA salts have significantly lower *T*_d_ than the NTf_2_^−^ salts. Fig. 2 illustrates the trends for the 10 °C data only. One to three methyl groups doesn't have a significant impact except for 4b for which the onset temperature is significantly lower at 279 °C. This is odd since 4a has a *T*_d_ of 331 °C.

### 2.4 Density

**Fig. 3:**
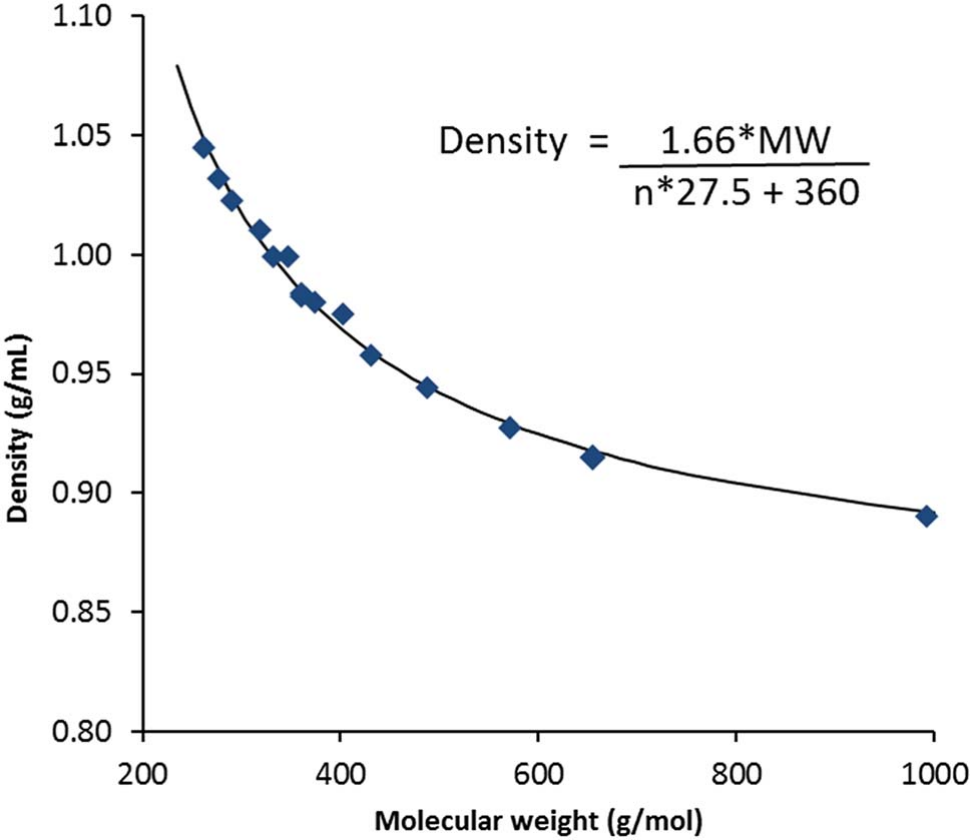
Density at 20 °C *versus* molecular weight for TDAC DCA salts.

Density data were determined from 20–90 °C where possible; the results for the 20 °C and 50 °C data are given in Table 2 and molar volumes are provided in the ESI.† Unlike melting points, viscosities and conductivities, *vide infra*, densities for TDAC DCA salts are effectively independent of cation shape and depend only on the cation size (or mass). Fig. 3 shows a plot of densities at 20 °C *versus* the MW of the cations for the DCA salts. At long chain lengths, densities must approach that of very low density polyethylene (VLDPE), 0.88–0.915 g mL^−1^, as the cation core and anions are increasingly diluted. The equation on Fig. 3 describes the fitted curve. This is made up of a volume for the permethyl salt 3a of 360 Å^3^ and a factor of 27.5 Å^3^ per CH_2_ group added (*n*) to this core. This CH_2_ volume agrees with that obtained by Ye and Shreeve for ILs of 28 Å^3^ and what we found for the NTf_2_^−^ salts of 27.7 Å^3^.^13,18^ We estimated a volume of 280 Å^3^ for the [C_3_(NMe_2_)_3_]^+^ cation in TDAC NTf_2_^−^ ILs which gives us a volume for DCA of 80 Å^3^. This is somewhat smaller than the 86 Å^3^ deduced by Ye and Shreeve.^18^

**Table 2:** MW, cation hydrodynamic radius, and selected density, viscosity, and conductivity data for TDAC DCA ILs

	MW (g mol^−1^)	*r* ^+^ at^a^ 20 °C (Å)	Density		Viscosity		Conductivity	
			20 °C	50 °C	20 °C	50 °C	20 °C	50 °C
[C_3_(NEt_2_)_3_]DCA (3b)	318.47	4.74	1.010	0.992	64.2	19.9	4.69	12.0
[C_3_(NPr_2_)_3_]DCA (3c)	402.63	5.27	0.975	0.957	107^b^	21.4^b^	—	—
[C_3_(NBu_2_)_3_]DCA (3d)	486.79	5.71	0.944	0.926	293	60.6	0.624	2.89
[C_3_(NPent_2_)_3_]DCA (3e)	570.95	6.09	0.927	0.909	308	60.9	0.295	1.44
[C_3_(NHex_2_)_3_]DCA (3f)	655.12	6.42	0.915	0.897	332	69.4	0.161	0.62
[C_3_(NDec_2_)_3_]DCA (3g)	991.76	7.51	0.890	0.872	554	97.5	0.022	0.13
[C_3_(NBuMe)_3_]DCA (4a)	360.55	5.02	0.983	0.964	101	24.1	1.92	6.35
[C_3_(NMe_2_)_2_(NPrMe)]DCA (8a)	262.36	4.31	1.045	1.025	107	25.1	3.17	12.4
[C_3_(NMe_2_)_2_(NBuMe)]DCA (8b)	276.39	4.43	1.032	1.014	67.4	17.2	4.38	15.1
[C_3_(NEt_2_)_2_(NBuMe)]DCA (9a)	332.49	4.84	0.999	0.981	73.7	20.8	3.50	9.50
[C_3_(NEt_2_)_2_(NHexMe)]DCA (9b)	360.55	5.02	0.984	0.966	86.2	23.4	2.77	8.82
[C_3_(NEt_2_)_2_(NMe_2_)]DCA (10a)	290.41	4.53	1.023	1.004	58.4	17.9	4.64	12.4
[C_3_(NEt_2_)_2_(NBu_2_)]DCA (10b)	374.58	5.10	0.980	0.962	105	27.4	2.20	6.703
[C_3_(NEt_2_)_2_(NHex_2_)]DCA (10c)	430.68	5.42	0.958	0.939	131	32.0	1.03	3.641

a Based on NTf_2_^−^ density data, see ref. 13 for details.

b Calculated based on 60–90 °C data.

At 20 °C and *n* = ∞, a density of 0.847 g mL^−1^ is calculated for TDAC DCA salts compared to 0.842 g mL^−1^ for the NTf_2_^−^ salts, effectively this is “liquid polyethylene”.

The densities of ILs have a linear dependency on temperature and can be well-fitted by the equation *ρ* = *a* − *bT*. Parameter a represents a theoretical density at 0 K. Pleasingly, a plot of parameter a *versus* cation MW can also be fit to a similar equation as the density data obtained at 20 °C (ESI, Fig. 1S†). In this case, we calculate a volume for each CH_2_ group at 0 K of 23 Å^3^ while the volume of 3a at 0 K was found to be 309 Å^3^. This gives a “free volume” of 51 Å^3^ for 3a at 20 °C and 4.5 Å^3^ per CH_2_. Parameter a for “liquid polyethylene” (density at 0 K and *n* = ∞) is 1.015 g mL^−1^.

Density parameter *b* represents the temperature dependency of the density. This parameter is rarely commented on, however, this parameter can also fit, although not as well, by an equation of the type used for the density at 20 °C and parameter a (ESI, Fig. 2S†). This illustrates a decrease in temperature dependence with MW. The combination of the equations for *a* and *b* allow us to derive a temperature-dependent equation for all TDAC DCA salts (eqn (1)).1
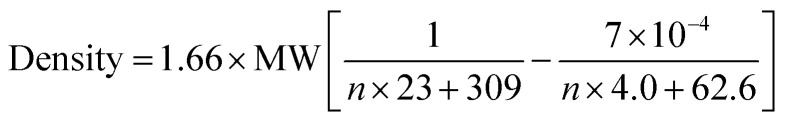


**Fig. 4:**
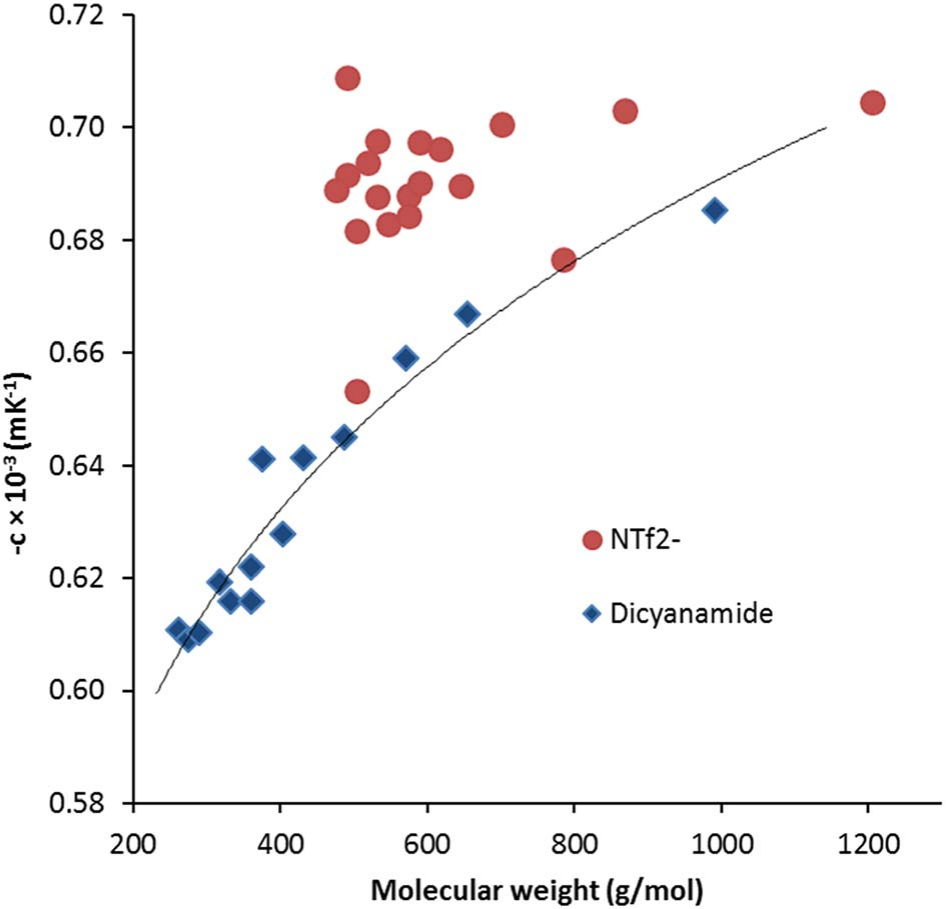
Thermal expansion coefficient *versus* molecular weight for DCA and NTf_2_^−^ TDAC salts. The trendline for the DCA salts is indicative only.

The thermal expansion coefficient *α*_p_ can be obtained from the slope of a plot of ln(*ρ*) *versus T*, *i.e.* −[∂ ln (*ρ*)/∂*T*]_P_ = −*c*. Although *α*_p_ can vary with temperature,^19^ and ln(*ρ*) *versus T* can be fit with quadratic or cubic functions, we have not done so due to the limited temperature range. The values of *α*_p_ range from 0.605 × 10^−3^ K^−1^ to 0.685 × 10^−3^ K^−1^ which is noticeably lower than the typical values for NTf_2_^−^ salts of 0.680–0.705 × 10^−3^ K^−1^. Fig. 4 illustrates a general increase with MW as well as the comparison with the NTf_2_^−^ salts. The DCA salts here are similar to values found for other ILs: phosphonium ILs (0.575–0.692 × 10^−3^ K^−1^),^20^ imidazolium ILs (0.579–0.705),^19,21^ and pyridinium ILs (0.530–0.543).^21^

### 2.5 Viscosity

**Fig. 5:**
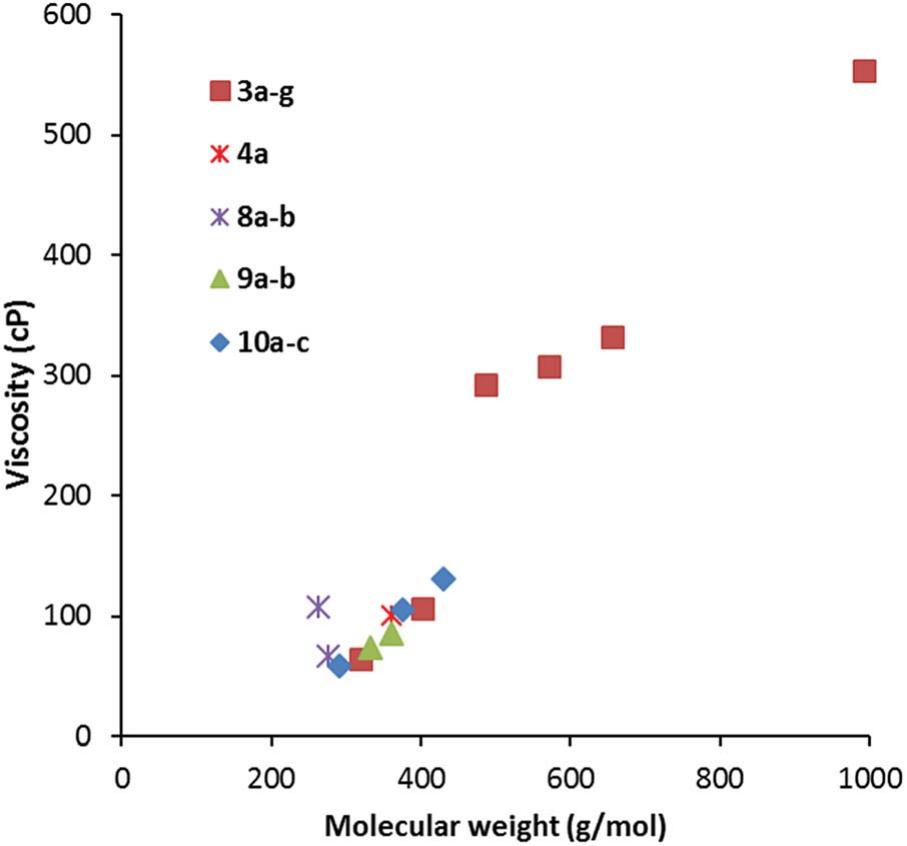
Viscosity at 20 °C for TDAC DCA salts.

**Fig. 6:**
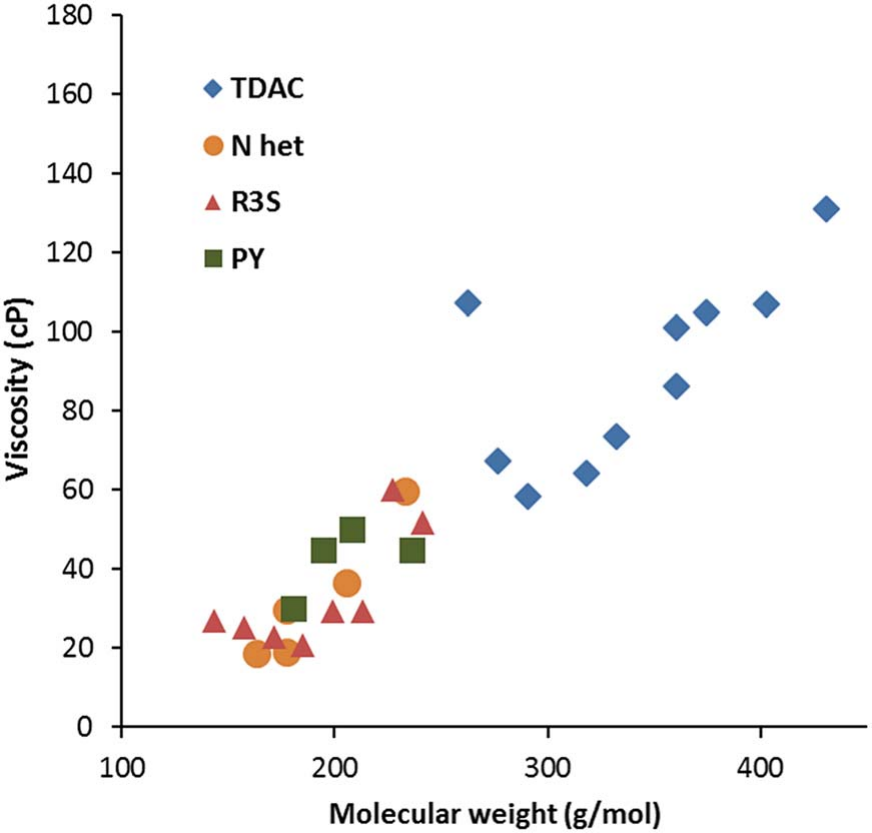
Viscosity at 20 °C for dicyanamide salts (N Het = imidazolium; R3S = sulfonium; PY = pyridinium).

Viscosity data were collected from 20–90 °C where possible; the results for the 20 °C and 50 °C data are given in Table 2. As is to be expected, viscosity generally increases with MW, as shown in Fig. 5 for viscosity at 20 °C. The data is colour-coded by symmetry class, however, it seems that symmetry has little effect except for a couple of cases: the propyl salt 8a appears to be an outlier in Fig. 5 and 6 with a much higher viscosity than the butyl derivative 8b. The hexabutyl salt 3d also seems higher than the other *D*_3h_ salts.

Fig. 6 compares the viscosity of the low MW salts to those of other cations at 20 °C. Generally, the TDAC salts have lower viscosities relative to their MW and this might be attributed to relatively weak cation–anion interactions. A similar effect was observed for the NTf_2_^−^ salts.^13^

The viscosity data was fit to both the Arrhenius (*η* = *A*_exp_(*E*_a_/*RT*)) and Vogel–Fulcher–Tammann (VFT, eqn (2)) equations (also, *D* = *B*/*T*_0_); these parameters are given in the ESI.† There are not many obvious trends: *E*_a_ tends to increase with MW; the values of *D*, a measure of the deviation from Arrhenius behaviour, lie in the range 4–10 which is typical for “fragile” liquids.^22^ A “fragility plot” of log(viscosity) *versus T*_g_/*T* (ESI, Fig. 4S†) similarly shows that these materials are typical of fragile ILs.2
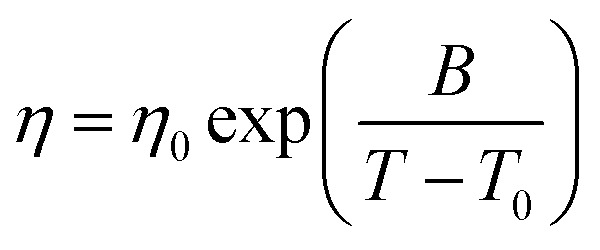


### 2.6 Conductivity

**Fig. 7:**
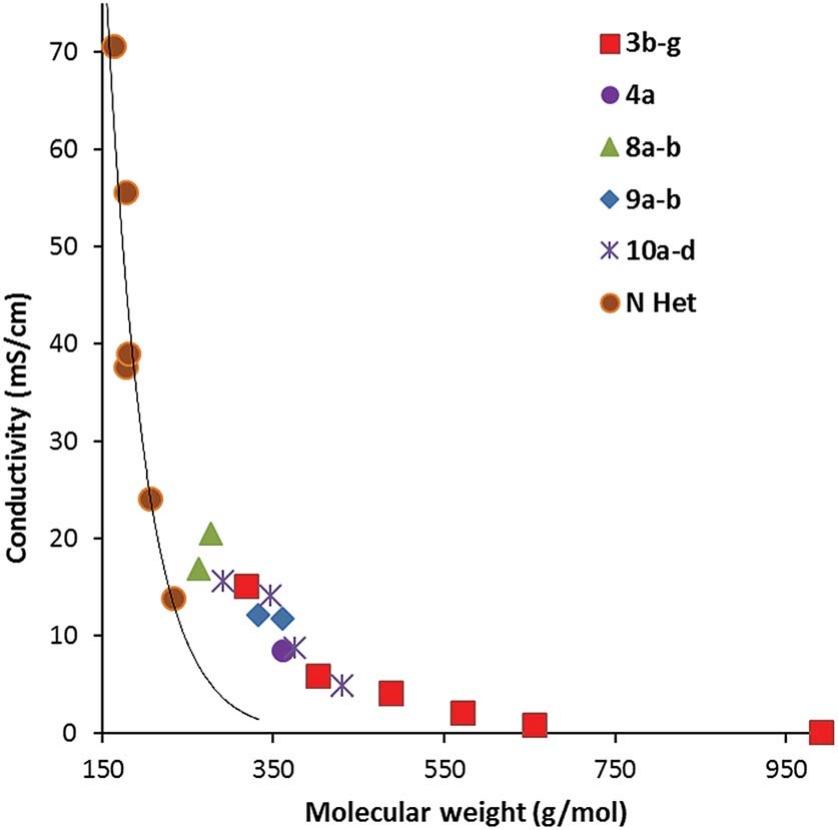
Conductivity at 60 °C for TDAC DCA ILs and some imidazolium^23^ (N Het) ILs.

Conductivity data was also collected from 20–90 °C where possible; results at 20 °C and 50 °C are given in Table 2. Fig. 7 illustrates the data at 60 °C for which a strong trend of decreasing conductivity with MW is observed. Although, as expected from the high viscosity of 8a, compared to 8b, it has a significantly lower conductivity than 8b. Interestingly, although the imidazolium DCA ILs generally have a higher conductivity, this is due to their lower MWs: Fig. 7 shows that the trendline for the imidazolium ILs lies well below that of the TDAC ILs. A plot of log(conductivity) *versus* MW gives a reasonably straight line (ESI, Fig. 5S†).

### 2.7 Ionicity

**Fig. 8:**
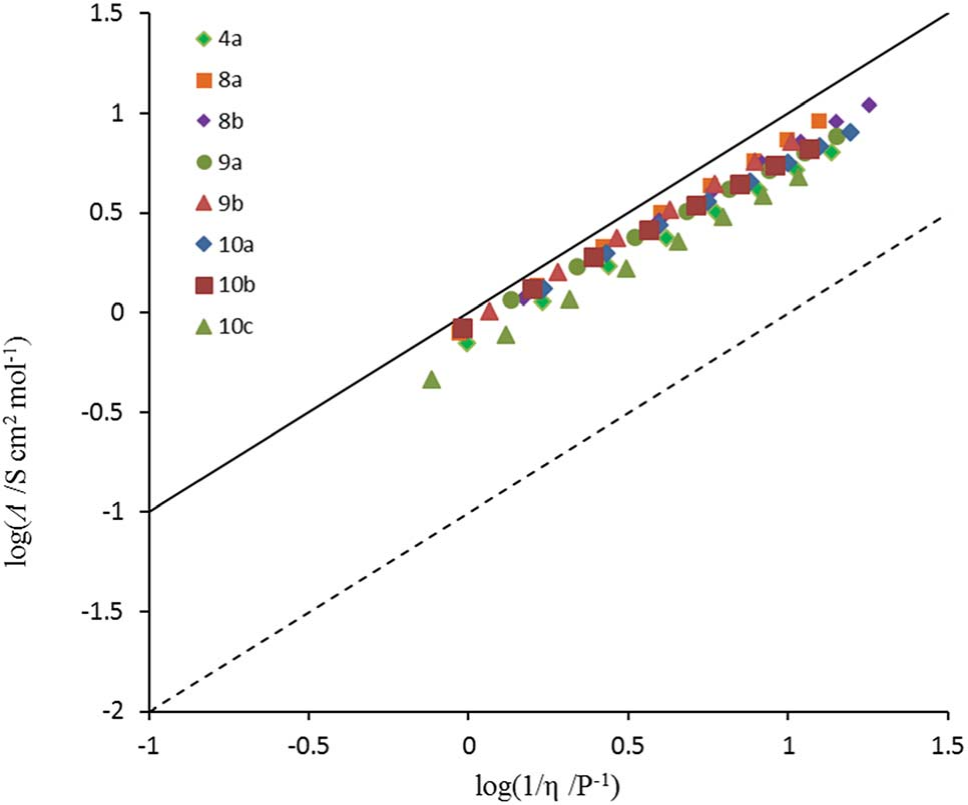
Walden plot for TDAC DCA salts (excludes (3b–g)).

**Fig. 9:**
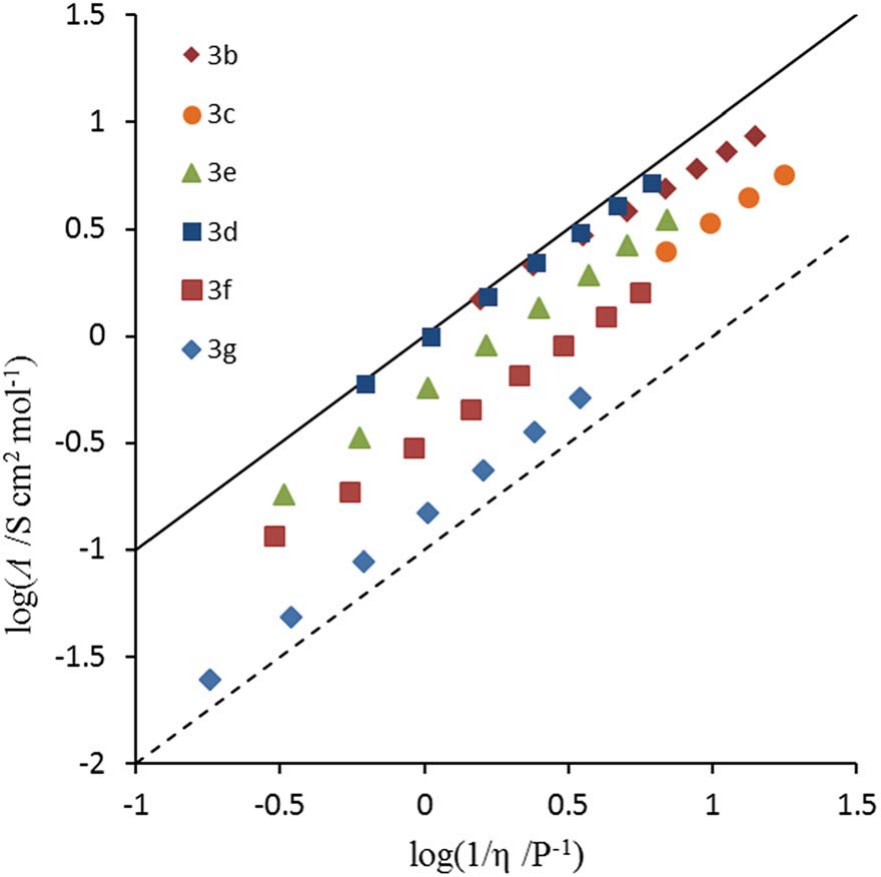
Walden plot for *D*_3h_-symmetric (3b–g) TDAC DCA ILs.

Conductivity and viscosity are linked through Walden's rule: *Λη* = *k* (in which *k* is a temperature-dependent constant, the Walden product). Walden plots, log(*Λ*) *versus* log(1/*η*), were used to investigate the ionicity of the ILs, with deviations from the ideal diagonal (1 M KCl(aq) line) being ascribed to the formation of ion pairs or aggregates (Fig. 8 and 9). The majority of ILs fall in a narrow band with only a small deviation from ideal, as measured by the average deviation Δ*W*. These would thus be considered as “good ILs”. The dihexyl salt 10c lies further from the ideal (Δ*W* = 0.28) than do the other non-*D*_3h_-symmetric cations (Δ*W* = 0.10–0.20), but as it did for the corresponding NTf_2_^−^ salt.^13^ However, it does not show the strongly non-linear plot that the NTf_2_^−^ salt does. The *D*_3h_-symmetric ILs (Fig. 9) show increasing deviation from ideal as size increases from 3d to 3g (Δ*W* = 0.04, 0.27, 0.50 and 0.84 for 3d–g, respectively). The same effect was observed for the corresponding NTf_2_^−^ salts. Curiously, the hexapropyl salt 3c (Δ*W* = 0.46) has a greater deviation than 3d and 3e, and the hexabutyl salt 3d has a remarkably small deviation of 0.04.

### 2.8 Miscibility and solubility

The miscibility and solubility properties of the salts were investigated at 25 °C. The results are given in Table 3 in order of increasing MW. DCA salts are more hydrophilic than NTf_2_^−^ salts. However, the DCA salts are miscible with water for only small MW (<*ca.* 315 g mol^−1^); they are immiscible for MW > *ca.* 400 g mol^−1^ and partially miscible for intermediate MWs. All of the salts were found to be soluble or miscible in MeOH, EtOH, CH_2_Cl_2_ and EtOAc. The notable exception being the insolubility in everything of 4b which has three C_18_ chains. That contrasts significantly with the similarly-sized 3g with six C_10_ chains that is soluble in everything except water. Most compounds are at least partially miscible in toluene, the small and symmetric 3a and 10a salts, as well as 4b, being the exceptions. As expected, larger alkyl chains favour miscibility in the non-polar solvents diethylether and hexane. Partial miscibility starts at about 320 g mol^−1^ in diethylether and 430 g mol^−1^ in hexane. As was found with the NTf_2_^−^ salts, greater symmetry decreases solubility/miscibility whereas increased flexibility increases solubility/miscibility. Thus 5 (with inflexible isopropyl groups) and *D*_3h_-symmetric 3c are insoluble in diethylether and hexane, and *C*_s_-symmetric 9b (with a flexible hexyl chain) is soluble in hexane despite a relatively small MW.

**Table 3:** Miscibility and solubility properties of TDAC DCA salts at 25 °C^a^

Compound	MW	Water	MeOH/EtOH/CH_2_Cl_2_/EtOAc	Toluene	Et_2_O	Hexane
[C_3_(NMe_2_)_3_]DCA (3a)	234	Y	Y	I	I	I
[C_3_(NMe_2_)_2_(NPrMe)]DCA (8a)	262	Y	Y	≥80% IL	N	N
[C_3_(NMe_2_)_2_(NBuMe)]DCA (8b)	276	Y	Y	≥50% IL	N	N
[C_3_(NEt_2_)_2_(NMe_2_)]DCA (10a)	290	Y	Y	N	N	N
[C_3_(NEt_2_)_3_]DCA (3b)	318	≥60% IL	Y	≥50% IL	≥50% IL	N
[C_3_(NEt_2_)_2_(NBuMe)]DCA (9a)	332	≥70% IL	Y	≥50% IL	≥50% IL	N
[C_3_(NBuMe)_3_]DCA (4a)	361	≥90% IL	Y	≥50% IL	≥50% IL	N
[C_3_(NEt_2_)_2_(NHexMe)]DCA (9b)	361	≥80% IL	Y	≥30% IL	≥50% IL	≥80% IL
[C_3_(NEt_2_)_2_(NBu_2_)]DCA (10b)	375	N	Y	≥50% IL	≥70% IL	N
[C_3_(NPr_2_)_3_]DCA (3c)	403	P	Y	P	I	I
[C_3_(N(i-Pr)_2_)_2_(NBu_2_)]DCA (5)	431	I	Y	P	I	I
[C_3_(NEt_2_)_2_(NHex_2_)]DCA (10c)	431	N	Y	Y	≥50% IL	≥70% IL
[C_3_(NBu_2_)_3_]DCA (3d)	487	N	Y	Y	≥50% IL	≥60% IL
[C_3_(NPent_2_)_3_]DCA (3e)	571	N	Y	Y	Y	≥60% IL
[C_3_(NHex_2_)_3_]DCA (3f)	655	N	Y	Y	Y	≥50% IL
[C_3_(StMe)_3_]DCA (4b)	950	I	I	I	I	I
[C_3_(NDec_2_)_3_]DCA (3g)	992	N	Y	Y	Y	Y

a I = insoluble; N = immiscible liquid; Y = soluble or miscible; P = partial miscibility.

### 2.9 X-ray crystallography

**Fig. 10:**
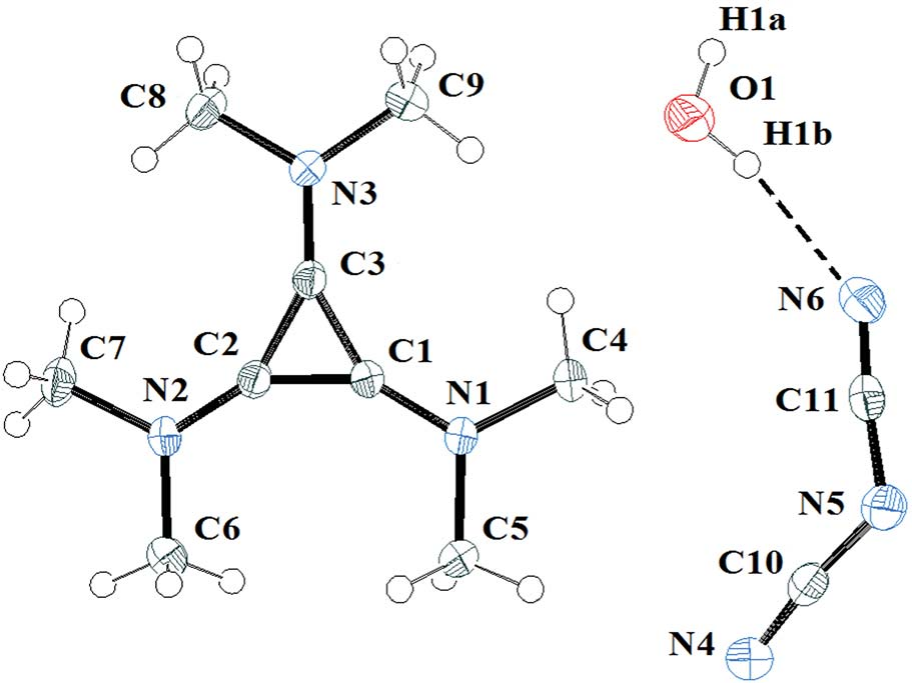
ORTEP of 3a·H_2_O illustrating the labelled asymmetric unit. Selected bond distances (Å): C1–C2 1.3801(13); C2–C3 1.3813(13); C1–C3 1.3833(13); C1–N1 1.3274(12); C2–N2 1.3305(12); C3–N3 1.3235(13).

The salts 3a and 3c were investigated by X-ray diffraction. Salt 3a was crystallised in air and consequently found to have one equivalent of water solvate. It packs in the *C*2/*c* space group with one independent cation, anion and water solvate in each unit cell. The DCA and water molecules form hydrogen-bonded chains that are parallel to chains of cations in the same layer. The layers above and below have the chains of cations above and below the chains of DCA and water. Fig. 10 illustrates the asymmetric unit with the atomic labelling scheme. The C–C distances range 1.3801(13)–1.3833(13) Å with an average of 1.382 Å. The exocyclic C–N distances range 1.3235(13)–1.3305(13) Å with an average of 1.327 Å while the N–Me distances range 1.4544(12)–1.4595(12) Å with an average of 1.457 Å. At least 16 salts containing [C_3_(NMe_2_)_3_]^+^ have been crystallographically characterised and the distances found here for the DCA salt are typical.^10,23^

Each cation has a DCA anion above and below with the shortest distances being C1⋯N4 at 3.305(1) Å and C3⋯N4 at 3.310(1) Å as well as a number of other inter-ion distances of less than 3.5 Å.

**Fig. 11:**
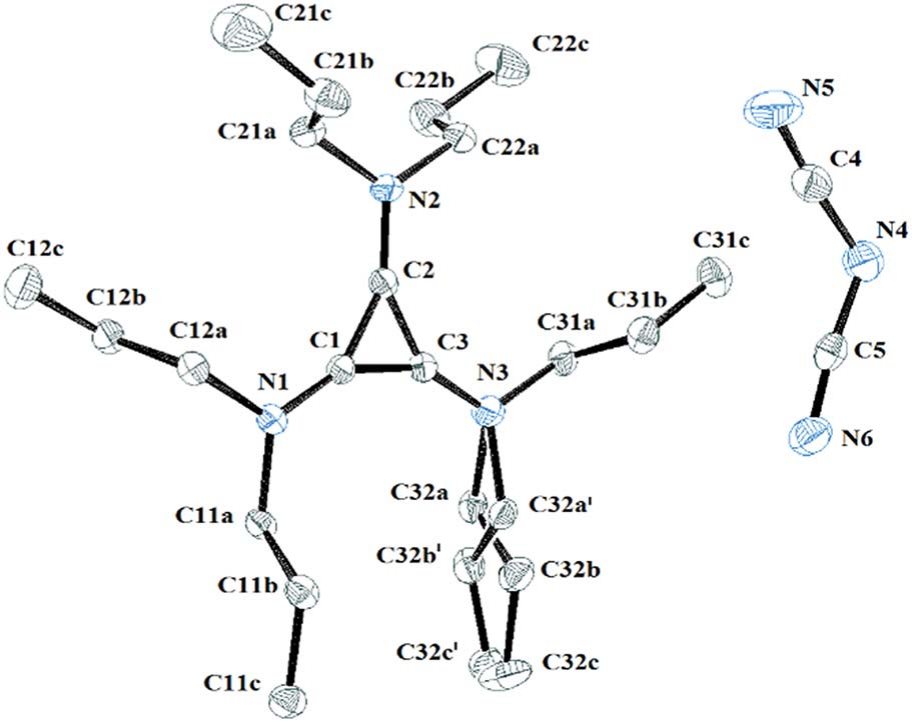
ORTEP of 3c illustrating the labelled asymmetric unit. Selected bond distances (Å): C1–C2 1.3801(18); C2–C3 1.3752(19); C1–C3 1.3738(19); C1–N1 1.3301(17); C2–N2 1.3286(17); C3–N3 1.3266(18).

**Fig. 12:**
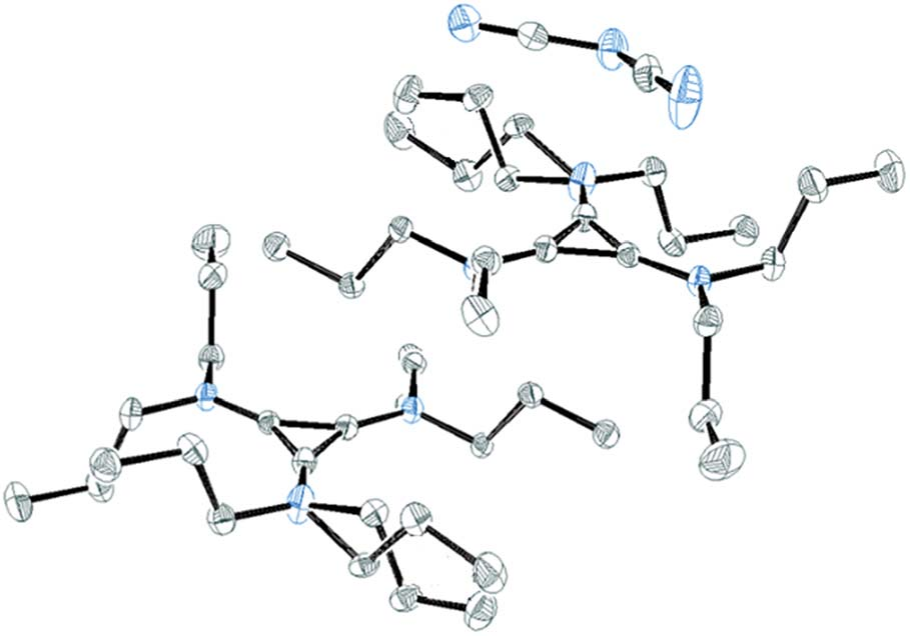
ORTEP of 3c illustrating a cation sandwiched between a DCA anion and a propyl group. The two cations are related *via* a *C*_2_ rotation axis.

Salt 3c packs in the *C*2/*c* space group with one independent cation and anion in the unit cell. Fig. 11 shows the two ions with the atomic labelling scheme. Each cation is sandwiched between a propyl group from another cation and a DCA anion that is parallel to the cyclopropenium plane (Fig. 12). The closest cyclopropenium ring to DCA distance is C3–N4 at 3.332 Å which is very similar to a distance of 3.351(2) Å for the distance between two positively-charged cyclopropenium cations in [C_3_(NEt_2_)_3_]I.^12^

One propyl group is disordered such that the attached N atom is non-planar. It is not clear why this would be the case, although it should be noted that the trisdiisopropyl isomer has one pyramidal N atom due to steric strain and a low rotational barrier of the amino groups in TAC cations.^25^ However, in that case, the C_3_N_3_ core is significantly asymmetric due to the incomplete delocalisation, whereas in 3c the C_3_N_3_ core is still close to *D*_3h_-symmetric.

## 3. Conclusions

We have prepared a series of TDAC DCA salts for comparison with a similar series of NTf_2_^−^ salts. Like those salts, the melting points can be rationalised by consideration of MW, symmetry and conformational flexibility. TGA studies found that dimethylamino groups generally decrease thermal stability. Density was similarly found to be strongly dependent on MW and temperature, but not symmetry. Density fitting parameters *a* and *b* are strongly correlated with MW, as is the thermal expansion coefficient *α*_P_ (which was not correlated in the NTf_2_^−^ salts). Viscosity appears to be less dependent on symmetry than in the NTf_2_^−^ salts and, similarly, conductivity is essentially MW dependent only. A Walden plot revealed that most of the ILs can be classified as “good ILs” with only the larger ILs showing deviations from ideality. The DCA salts are more hydrophilic than the NTf_2_^−^ salts, but even so, only the smaller cations are soluble/miscible in water. Solubility/miscibility in non-polar solvents was, like the NTf_2_^−^ salts, found to be largely dependent on MW, but also subtly dependent on symmetry and conformational flexibility. Only the largest cation, 3g, is fully miscible with hexane. The extensive characterisation of a second comparative anion series of TDAC salts will make it easier to predict properties of TDAC salts with other anions and thus ease their tunability.

## 4. Experimental

All operations were performed using standard Schlenk techniques with a dinitrogen atmosphere in order to reduce exposure to water. ^1^H-, ^13^C{^1^H}-NMR spectra were collected on a Varian Unity-300 operating at 300 and 75 MHz, respectively, an Agilent DD2-400MR operating at 400 and 100 MHz, respectively, or on a Varian INOVA-500 operating at 500 and 126 MHz, respectively, in CDCl_3_, referenced to residual solvent peaks. Electrospray mass spectrometry was carried out on a Micromass LCT, with samples dissolved in acetonitrile. Water contents were determined by Karl Fischer titration using a Metrohm 831 KF coulometer. Chloride contents were determined using an AutolabEco Chemie, with associated GPES software, under a dinitrogen atmosphere. The electrodes were either a glassy carbon (3 mm diameter) or platinum (1 mm diameter) working electrode, a platinum wire counter electrode and a silver reference electrode. Microanalysis was performed by Campbell Microanalytical Laboratory, Dunedin. NaDCA, pentachlorocyclopropane (Acros), dimethyl sulfate, diisopropylamine, dibutylamine (Koch-Light), butylmethylamine, and propylamine, were used as obtained commercially. The following salts were prepared by previously published methods: [C_3_(NR_2_)_3_]DCA (R = Et (3b)^14^ and Bu (3d))^6^; [C_3_(NEt_2_)_2_(NRMe)]DCA (R = Bu (9a), Hex (9b)); and [C_3_(NEt_2_)_2_(NR_2_)]DCA (R = Me (10a), Bu (10b), Hex (10c)).^14^ The starting materials (Et_2_N)_2_C_3_O,^14^ [C_3_(NMe_2_)_3_]Cl (1a),^13^ [C_3_(NPr_2_)_3_]Cl,^6^ [C_3_(NPent_2_)_3_]Cl,^13^ [C_3_(NHex_2_)_3_]Cl,^13^ [C_3_(NDec_2_)_3_]Cl,^13^ [C_3_(NBuMe)_3_]Cl,^6^ [C_3_(NC_18_Me)_3_]Cl^13^ and [C_3_(NMe_2_)_2_(OMe)]MeSO_4_ (7)^13^ were prepared by previously described procedures. The ESI† contains the full synthesis and characterisation details of the TDAC DCA salts: 3a, 3c, 3e, 3f, 3g, 4a, 4b, 5, 8a, and 8b.

DSC was performed on a Perkin Elmer Q100: samples of mass 5–20 mg were sealed in a vented aluminium pan and placed in the furnace with a 50 mL min^−1^ nitrogen stream; the temperature was raised at 10 °C min^−1^. TGA data were collected on dried samples using a TA Instruments SDT Q600 at 10 °C min^−1^ after further drying at 100 °C for one hour in the instrument. Density measurements were carried out on an Anton Parr DMA 5000 instrument, an oscillating U-tube density meter, from 20 to 90 °C in 10 °C steps. Viscosities were measured on a Brookfield-Wells cone-and-plate viscometer operating at 0.005–0.2 s^−1^ rotation speed range. Conductivities were measured using a Schott LF4100+ probe and an impedance bridge conductivity meter. The instrument was calibrated with 0.1 mol L^−1^ KCl solution.

Solubility and miscibility studies were carried out by taking 0.5 mL of sample and adding step-wise 10 × 0.05 mL of solvent followed by 9 × 0.5 mL of solvent. After each addition of solvent the sample was mixed and allowed to equilibrate at 25 °C to determine whether the sample was miscible or immiscible. In the case of solid samples, a 0.1 g sample was taken and 2.5 mL of solvent was added and the sample was equilibrated at 25 °C. In some cases, the solid sample was observed to form two immiscible liquid layers. Water contents of water-saturated ILs were measured by Karl-Fischer titration after equilibration at 25 °C for 24 hours followed by centrifugation.

X-ray crystallography: single crystals of 3a·H_2_O and 3c formed in the neat liquid. A suitable crystal of each was selected and mounted on a SuperNova, Dual, Cu at zero, Atlas diffractometer. Using Olex2,^26^ the structures were solved with the XS structure solution program^27^ using Direct Methods and refined with the XL refinement package^27^ using Least Squares minimisation. Crystal data and structure refinement and structural details are given in the ESI† along with the atom numbering schemes.

## Conflicts of interest

The authors declare that they have no conflict of interest.

## Supplementary Material

RA-008-C8RA05558K-s001

RA-008-C8RA05558K-s002
