# Supplementary material for: Synthesis and physical properties of tris(dialkylamino)cyclopropenium dicyanamide ionic liquids
Source: RSC Adv. 2018 Aug 7;8(50):28313–22. doi: 10.1039/c8ra05558k (PMC9083934; doi:10.1039/c8ra05558k)
Supplement: RA-008-C8RA05558K-s001 [file RA-008-C8RA05558K-s001.pdf]

Electronic Supplementary Material (ESI) for RSC Advances.
This journal is © The Royal Society of Chemistry 2018

Supplementary material

Synthesis and physical properties of tris(dialkylamino)cyclopropenium dicyanamide ionic liquids

Owen J. Curnow,* Matthew I. J. Polson, Kelvin J. Walst and Ruhamah Yunis

School of Physical and Chemical Sciences, University of Canterbury, Private Bag 4800, Christchurch 8041, New Zealand. E-mail: owen.curnow@canterbury.ac.nz; Tel: +64 3 369 4239.

Contents | **Page**

**Starting materials**	2
**Synthesis of TDAC DCA salts**	2
Table 1S. Density data for TDAC DCA ionic liquids.	5
Table 2S. Density fitting parameters for TDAC DCA ILs.	5
Table 3S. Molar volumes (mL mol^{-1}) for TDAC DCA ILs.	6
**Fig. 1S.** Density parameter a versus cation molecular weight for TDAC DCA ILs.	6
**Fig. 2S.** Density parameter b versus cation molecular weight for TDAC DCA ILs.	7
**Fig. 3S.** d (the intercept of ln(ρ) *versus* T plots) *versus* cation molecular weight for TDAC DCA ILs.	7
Table 4S. Viscosity data for TDAC DCA ILs.	8
Table 5S. Viscosity fit parameters for TDAC DCA ILs.	8
**Fig. 4S.** A fragility plot for the salts with observed glass transition temperatures.	9
Table 6S. Viscosities of non-TDAC cations with DCA.	9
Table 7S. Conductivity data for TDAC DCA ILs.	10
Table 8S. Conductivity fit parameters for TDAC DCA ILs.	10
**Fig. 5S.** Plot of Log(conductivity) versus MW with a linear fit.	11
Table 9S. Walden parameters for TDAC DCA ILs	11
**X-ray crystallography**	12
Table 10S. Crystallographic data for **3a**.H_2O and **3c.**	12
Table 11S. Bond lengths (Å) and angles (°) for [$C_3(NMe_2)_3$]DCA.H_2O (**3a**.H_2O).	13
**Fig. 6S.** Illustration of one layer of **3a**.H_2O and the cation environment.	13
Table 12S. Bond lengths for [$C_3(NPr_2)_3$]DCA (**3c**).	14
Table 13S. Bond angles for [$C_3(NPr_2)_3$]DCA (**3c**).	14
**References**	14

Starting materials

Bis(diisopropylamino)dibutylaminocyclopropenium chloride, $[C_3(N^iPr_2)_2NBu_2]Cl$

C_3Cl_5H (5.17 g, 24.1 mmol) was stirred at 0 °C in dry dichloromethane (75 mL). HN^iPr_2 (11.9 g, 118 mmol) added dropwise over 1 h followed by stirring for 2 h at 0 °C and then overnight at ambient temperature. HN^nBu_2 (12.3 g, 95.0 mmol) was added dropwise over 1 h, followed by stirring for 2 h at 0 °C, then overnight at ambient temperature, then heated to reflux for 4 h. The dichloromethane was removed *in vacuo* and dilute NaOH added. The aqueous solution was washed with diethyl ether (3 x 75 mL). The solution was neutralized with aqueous HCl and the product extracted with dichloromethane (3 x 125 mL). Drying *in vacuo* yielded a colourless solid (5.77 g, 60%). ^1H NMR (300 MHz, $CDCl_3$): 3.79 (spt, $^3J_{HH}$ = 13.8, 7.0 Hz, 4H, $NCH(CH_3)_2$), 3.36 (t, $^3J_{HH}$ = 7.9 Hz, 4H, NCH_2), 1.53 (m, 4H, NCH_2CH_2), 1.30 (d, $^3J_{HH}$ = 7.0 Hz, 24H, $NCH(CH_3)_2$), 1.23 (m, 4H, $NCH_2CH_2CH_2$), 0.89 (t, $^3J_{HH}$ = 7.90 Hz, 6H, CH_2CH_3). ^{13}C NMR (75 MHz, $CDCl_3$): 118.46 (C_1), 117.49 (C_2), 51.57 (NCH_2), 51.50 ($NCH(CH_3)_2$), 30.77 (NCH_2CH_2), 21.99 ($NCH(CH_3)_2$), 19.44 ($N(CH_2)_2CH_2CH_3$), 13.67 ($N(CH_2)_3CH_3$). ES^+ m/z 364.3690 (100%, M^+). Found: C, 67.83; H, 11.48; N, 10.50%. Calc. for $C_{23}H_{46}N_3Cl.0.40H_2O$: C, 67.83; H, 11.58; N, 10.32%.

Bis(diethylamino)methoxycyclopropenium methylsulphate, $[C_3(NEt_2)_2OMe]MeSO_4$. Dried bis(diethylamino)cyclopropenone (1.14 g, 6 mmol) was stirred with Me_2SO_4 (0.98 mL, 8 mmol) for 2 h in an inert atmosphere. Solvent was removed *in vacuo.* The mixture was washed with dry diethyl ether several times to remove excess Me_2SO_4 and cyclopropenone. This gave an orange viscous oil of $[C_3(NEt_2)(OMe)]MeSO_4$ (1.46 g, 70%). ^1H NMR (500 MHz, $CDCl_3$): δ 4.19 (s, 3H, OCH_3), 3.5 (s, 3H, CH_3SO_4), 3.43 (q, $^3J_{HH}$ = 7.3 Hz, 8H, NCH_2CH_3), 1.3 (t, $^3J_{HH}$ = 7.25 Hz, 12H, NCH_2CH_3).

Synthesis of TDAC DCA Salts

Tris(dimethylamino)cyclopropenium dicyanamide, $[C_3(NMe_2)_3]$DCA (3a). $[C_3(NMe_2)_3]Cl$ (4.35 g, 21 mmol) was stirred with NaDCA (5.68 g, 64 mmol) in water (50 mL) at 40 °C overnight. The product was extracted with chloroform (3 x 10 mL). The solvent was removed *in vacuo* to yield a yellow solid (2.7 g, 54%). ^1H, ^{13}C NMR and MS^+ as for $[C_3(NMe_2)_3]Cl$ with an additional peak in the ^{13}C NMR due to DCA. Found: C, 55.64; H, 7.93; N, 33.63%. Calc. for $C_{11}H_{18}N_6$ with 15% chloride salt: C, 55.89; H, 7.92; N, 33.58%.

Tris(dipropylamino)cyclopropenium dicyanamide, $[C_3(NPr_2)_3]$DCA (3c). $[C_3(NPr_2)_3]Cl$ (7.69 g, 20.7 mmol) was stirred with aqueous NaDCA (3.73 g, 41.9 mmol) in water (100 mL). The product was extracted with dichloromethane (50 mL) and washed with water (3 x 100 mL). Additional aqueous NaDCA (1.85 g, 20.8 mmol) was added and the organic layer was then washed with water (4 x 100 mL) and dried *in vacuo* to yield a yellow liquid (11.5 g, 85%) which slowly crystallized. ^1H, ^{13}C NMR and MS^+ as for $[C_3(NPr_2)_3]Cl$. Found: C, 67.19; H, 10.57; N, 19.77%. Calc. for $C_{23}H_{42}N_6.0.6H_2O$: C, 66.82; H, 10.53; N, 20.33%. Cl^- content: 314 ppm.

Tris(dipentylamino)cyclopropenium dicyanamide, $[C_3(NPent_2)_3]$DCA (3e). $[C_3(NPent_2)_3]Cl$ (4.13 g, 7.66 mmol) was stirred with aqueous NaDCA (3.8 g, 43.1 mmol) in water (100 mL). The product was extracted with diethyl ether (200 mL), and additional NaDCA (6.1 g, 44.9 mmol) and water (100 mL) was added. The diethyl ether solution was then washed with water (3 x 100 mL) and dried *in vacuo* to yield an orange liquid (3.69 g, 85%). ^1H, ^{13}C NMR and MS^+ as for $[C_3(NPent_2)_3]Cl$. Found: C, 73.26; H, 11.58; N, 14.64%. Calc. for $C_{35}H_{66}N_5.0.16H_2O$: C, 73.26; H, 11.65; N, 14.65%. H_2O content: 316 ppm. Cl^- content: 312 ppm.

Tris(dihexylamino)cyclopropenium dicyanamide, $[C_3(NHex_2)_3]$DCA (3f). $[C_3(NHex_2)_3]$Cl (3.85 g, 6.16 mmol) was stirred with NaDCA (2.5 g, 28.1 mmol) in water (50 mL). The product was extracted with dichloromethane (25 mL) and washed with water (50 mL). Additional NaDCA (2.5 g, 28.1 mmol) and water (100 mL) was added. Extracted with diethyl ether (100 mL), washed with water (4 x 100 mL) and dried *in vacuo* to yield an orange liquid (3.61 g, 89%). ^{1}H, ^{13}C NMR and MS$^+$ as for $[C_3(NHex_2)_3]$Cl. Found: C, 75.31; H, 12.25; N, 12.72%. Calc. for $C_{41}H_{78}N_6$: C, 75.17; H, 12.00; N, 12.83%. H_2O content: 272 ppm. Cl^- content: 1055 ppm.

Tris(didecylamino)cyclopropenium dicyanamide, $[C_3(NDec_2)_3]$DCA (3g). $[C_3(NDec_2)_3]$Cl (3.10 g, 3.23 mmol) was stirred with NaDCA (2.0 g, 22.5 mmol). Water (100 mL) was added and product extracted with diethyl ether (200 mL). Additional NaDCA (2.0 g, 22.5 mmol) and water (100 mL) was added. Diethyl ether solution was washed with water (3 x 100 mL) and dried *in vacuo* to yield an orange liquid (3.10 g, 97%). ^{1}H, ^{13}C NMR and MS$^+$ as for $[C_3(NDec_2)_3]$Cl. Found: C, 78.61; H, 12.88; N, 8.51%. Calc. for $C_{65}H_{126}N_4S_2O_4F_6$: C, 78.72; H, 12.81; N, 8.47%. H_2O content: 186 ppm. Cl^- content: 369 ppm.

Tris(butylmethylamino)cyclopropenium dicyanamide, $[C_3(NBuMe)_3]$DCA (4a). $[C_3(NBuMe)_3]$Cl (5.30 g, 16.1 mmol) was stirred with NaDCA (3.65 g, 41.0 mmol) in water (100 mL). The product was extracted with chloroform (50 mL) washed with water (3 x 50 mL)) and dried *in vacuo* to yield a yellow liquid (5.40 g, 93%). ^{1}H, ^{13}C NMR and MS$^+$ as for $[C_3(NBuMe)_3]$Cl with an additional peak due to DCA. Found: C, 66.33; H, 10.27; N, 23.19%. Calc. for $C_{20}H_{36}N_6$: C, 66.63; H, 10.06; N, 23.31%. H_2O content: 1139 ppm. Cl^- content: 156 ppm.

Tris(octadecylmethylamino)cyclopropenium dicyanamide, $[C_3(NC_{18}Me)_3]$DCA (4b). $[C_3(NC_{18}Me)_3]$Cl (4.00 g, 4.35 mmol) was stirred with NaDCA (1.2 g, 13.5 mmol) in water (100 mL). The product was extracted with dichloromethane (100 mL), washed with water (3 x 100 mL) and dried *in vacuo* to yield a yellow solid (3.30 g, 80%). ^{1}H, ^{13}C NMR and MS$^+$ as for $[C_3(NC_{18}Me)_3]$Cl. Found: C, 77.31; H, 12.90; N, 9.10%. Calc. for $C_{62}H_{120}N_6 0.75H_2O$: C, 11.31; H, 12.71; N, 8.72%.

Bis(diisopropylamino)dibutylaminocyclopropenium dicyanamide, $[C_3(N^iPr_2)_2NBu_2]$DCA (5). $[C_3(N^iPr_2)_2NBu_2]$Cl (0.300 g, 0.750 mmol) was stirred with NaDCA (0.350 g, 3.93 mmol) in water (50 mL). The product was extracted with chloroform (50 mL). Additional NaDCA (0.519 g, 5.83 mmol) in water (50 mL) was added. The chloroform was washed with water (3 x 50 mL) and dried *in vacuo* to yield a colourless solid (0.25 g, 78%). ^{1}H, ^{13}C NMR and MS$^+$ as for $[C_3(N^iPr_2)_2NBu_2]$Cl. Found: C, 69.67; H, 10.76; N, 19.64%. Calc. for $C_{25}H_{46}N_4S_2O_4F_6$: C, 69.72; H, 10.77; N, 19.51%. Cl^- content: 1176 ppm.

Bis(dimethylamino)propylmethylaminocyclopropenium dicyanamide, $[C_3(NMe_2)_2(NPrMe)]$DCA (8a). $[C_3(NMe_2)_2(OMe)]MeSO_4$ (4.7 g, 18 mmol) was stirred with $H_2N(CH_2CH_2CH_3)$ (18 mL, 23 mmol) in dry dichloromethane (5 mL) for 2 h in an inert atmosphere. Dichloromethane was removed *in vacuo.* The mixture was in dissolved water and the product was extracted with chloroform:ethanol 2:1 (3 × 30 mL). The solvent was removed *in vacuo* to give $[C_3(NMe_2)_2(N(CH_2CH_2CH_3)H)]MeSO_4$ (3.51 g, 59%) as a light yellow oil, which was then stirred with NaDCA (2.79 g, 14 mmol) in H_2O (20 mL). The product was extracted with chloroform (3 × 30 mL). The solvent was removed *in vacuo* to give a yellow oil (3 g, 98%) of $[C_3(NMe_2)_2(N(CH_2CH_2CH_3)H)]$DCA. This was dried under vacuum overnight.
$[C_3(NMe_2)_2(N(CH_2CH_2CH_3)H)]$DCA (3 g, 10 mmol) was stirred with dry THF at −78 °C and *n*-BuLi (6.87 mL of 1.6 M, 11 mmol) was added drop wise in an inert atmosphere. Reaction mixture was stirred for 30 minutes and then allowed to warm to room temperature for another 30 minutes. Me_2SO_4 (1.66 mL, 13 mmol) was

then added and the solution was stirred for another 30 minutes. $LiMeSO_4$ was removed by filtration. THF was then removed *in vacuo*. The mixture was dissolved in water (50 mL) with NaDCA (1 g, 11 mmol) and the product was extracted with $CHCl_3$ (3 × 50 mL). The solvent was removed *in vacuo* to give a yellow oil (3 g, 96%). ^1H NMR ($CDCl_3$, 400 MHz): δ 3.32 (t, $^3J_{HH}$ = 8 Hz, 2H, NCH_2), 3.17 (s, 12H, NCH_3), 3.15 (s, 3H, NCH_3), 1.69 (m, 2H, NCH_2CH_2), 0.94 (t, $^3J_{HH}$ = 8 Hz, 3H, $NCH_2CH_2CH_3$). $^{13}C\{^1H\}$ NMR ($CDCl_3$, 100 MHz): δ 118.88 (CN), 117.71 (equivalent ring C), 117.51 (unique ring C), 57.15 (NCH_2), 42.49 ($N(CH_3)_2$), 40.30 (NCH_3), 21.06 (NCH_2CH_2), 10.95 ($NCH_2CH_2CH_3$). ESI MS: m/z 196.1808 (M^+). Anal. calcd for $C_{13}H_{22}N_6.0.75H_2O$: C, 56.60; H, 8.58; N, 30.46. Found: C, 56.96; H, 8.07; N, 30.33. With water content of 793 ppm.

Bis(dimethylamino)butylmethylaminocyclopropenium dicyanamide, $[C_3(NMe_2)_2(NBuMe)]$DCA (8b). $[C_3(NMe_2)_2(OMe)]MeSO_4$ (3.3 g, 13 mmol) was stirred with BuMeNH (1.9 mL, 17 mmol) in dry dichloromethane (5 mL) for 30 minutes in an inert atmosphere. Dichloromethane was removed *in vacuo.* The mixture was washed with dry diethyl ether several times to remove excess amine. The mixture was dissolved in water and the product was extracted with dichloromethane (3 ×30 mL). The solvent was removed *in vacuo* to give $[C_3(NMe_2)_2(NBuMe)]MeSO_4$ (3.27 g, 80%) as a brown oil which was then stirred with NaDCA (2.7 g, 123 mmol) in H_2O (20 mL). The product was extracted with chloroform (3×30 mL) and the solvent was then removed *in vacuo* to give a brown oil (2.66 g, 95%). ^1H NMR (400 MHz, $CDCl_3$): δ 3.33 (t, 2H, $^3J_{HH}$ = 8 Hz, NCH_2), 3.16 (s, 12H, NCH_3), 3.14 s, 3H, NCH_3), 1.63 (m, 2H, NCH_2CH_2), 1.33 (m, 2H, $NCH_2CH_2CH_2$), 0.93 (t, $^3J_{HH}$ = 8 Hz, 3H, $CH_2CH_2CH_3$). $^{13}C\{^1H\}$ NMR (100 MHz, CDCl3): δ 119.88 (CN), 117.79 (equivalent ring C), 117.52 (unique ring C), 55.44 (NCH_2), 42.51 ($N(CH_3)_2$), 40.31 (NCH_3), 29.80 (NCH_2CH_2), 19.83 ($NCH_2CH_2CH_2$), 13.72 ($NCH_2CH_2CH_2CH_3$). ESI MS: m/z 210.1964 (M^+). Anal. calcd for $C_{14}H_{24}N_6.0.4H_2O$: C, 59.29; H, 8.81; N, 29.64. Found: C, 58.94; H, 8.99; N, 29.13. With water content of 1482 ppm.

Table 1S. Density data for TDAC DCA ILs.

Compound	Density (g.cm^{-3})							
	20 °C	30 °C	40 °C	50 °C	60 °C	70 °C	80 °C	90 °C
$[C_3(NEt_2)_3]$DCA (**3b**)	1.010	1.004	0.998	0.992	0.985	0.979	0.973	0.967
$[C_3(NPr_2)_3]$DCA (**3c**)	–	–	–	–	0.951	0.945	0.939	0.933
$[C_3(NBu_2)_3]$DCA (**3d**)	0.944	0.938	0.932	0.926	0.920	0.914	0.908	0.902
$[C_3(NPent_2)_3]$DCA (**3e**)	0.927	0.921	0.915	0.909	0.903	0.897	0.891	0.885
$[C_3(NHex_2)_3]$DCA (**3f**)	0.915	0.909	0.903	0.897	0.891	0.885	0.879	0.873
$[C_3(NDec_2)_3]$DCA (**3g**)	0.890	0.884	0.878	0.872	0.866	0.860	0.854	0.849
$[C_3(NBuMe)_3]$DCA (**4a**)	0.983	0.977	0.970	0.964	0.958	0.952	0.947	0.941
$[C_3(NMe_2)_2(NPrMe)]$DCA (**8a**)	1.045	1.039	1.032	1.025	1.019	1.013	1.007	1.001
$[C_3(NMe_2)_2(NBuMe)]$DCA (**8b**)	1.032	1.026	1.020	1.014	1.007	1.002	0.995	0.989
$[C_3(NEt_2)_2(NBuMe)]$DCA (**9a**)	0.999	0.993	0.987	0.981	0.975	0.969	0.963	0.957
$[C_3(NEt_2)_2(NHexMe)]$DCA (**9b**)	0.984	0.978	0.972	0.966	0.960	0.954	0.948	0.942
$[C_3(NEt_2)_2(NMe_2)]$DCA (**10a**)	1.023	1.017	1.010	1.004	0.998	0.992	0.986	0.980
$[C_3(NEt_2)_2(NBu_2)]$DCA (**10b**)	0.980	0.974	0.968	0.962	0.955	0.949	0.943	0.937
$[C_3(NEt_2)_2(NHex_2)]$DCA (**10c**)	0.958	0.952	0.946	0.939	0.933	0.927	0.922	0.916

Table 2S. Density fitting parameters for TDAC DCA ILs

Compound	$-b \times 10^{-4}$	a	α_p $-c\times10^{-3}$	d
**3b**	6.123	1.1894	0.61941	0.19165
**3c**	5.989	1.1504	0.62790	0.15875
**3d**	5.953	1.1183	0.64493	0.13148
**3e**	5.973	1.1021	0.65911	0.11772
**3f**	5.962	1.0896	0.66694	0.10675
**3g**	5.958	1.0648	0.68532	0.08475
**4a**	5.980	1.1577	0.62198	0.16475
**8a**	6.315	1.2300	0.61084	0.22508
**8b**	6.181	1.2113	0.60912	0.21035
**9a**	6.025	1.1759	0.61584	0.18005
**9b**	5.929	1.1571	0.61587	0.16397
**10a**	6.110	1.2016	0.61027	0.20138
**10b**	6.145	1.1601	0.64110	0.16789
**10c**	6.006	1.1335	0.64137	0.14476

Table 3S. Molar volumes (mL mol^{-1}) for TDAC DCA ILs

Compound	MW	293 K	303 K	313 K	323 K	333 K	343 K	353 K	363 K
**3b**	318.47	315.28	317.23	319.20	321.18	323.18	325.19	327.21	329.25
**3c**	402.63	412.96	415.56	418.16	420.79	423.45	426.12	428.82	431.51
**3d**	486.79	515.65	518.99	522.33	525.71	529.10	532.52	535.98	539.49
**3e**	570.95	615.72	619.83	623.89	628.00	632.16	636.35	640.57	644.82
**3f**	655.12	715.96	720.74	725.51	730.36	735.25	740.19	745.16	750.18
**3g**	991.76	1113.85	1121.61	1129.35	1137.05	1144.85	1152.72	1160.66	1168.66
**4a**	360.55	366.97	369.21	371.52	373.85	376.19	378.54	380.90	383.28
**8a**	262.36	251.06	252.51	254.22	255.96	257.47	258.99	260.54	262.10
**8b**	276.39	267.82	269.38	270.97	272.57	274.46	275.83	277.77	279.46
**9a**	332.49	332.66	334.71	336.77	338.85	340.94	343.05	345.18	347.31
**9b**	360.55	366.58	368.83	371.11	373.40	375.71	378.04	380.37	382.72
**10a**	290.41	283.95	285.69	287.45	289.21	290.98	292.76	294.55	296.34
**10b**	374.58	382.19	384.62	387.08	389.56	392.07	394.60	397.15	399.72
**10c**	430.68	449.75	452.61	455.51	458.44	461.39	464.37	467.37	470.39

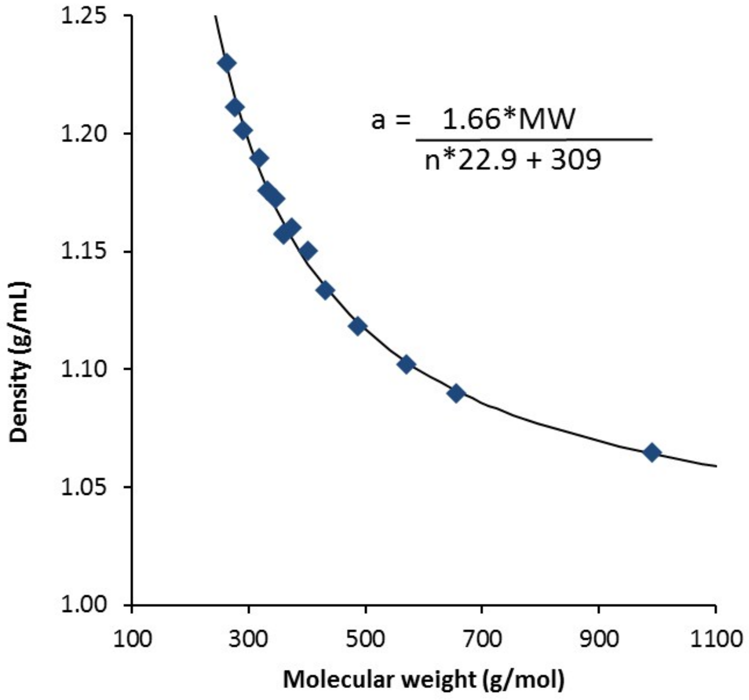

Fig. 1S. Density parameter a versus cation molecular weight for TDAC DCA ILs.

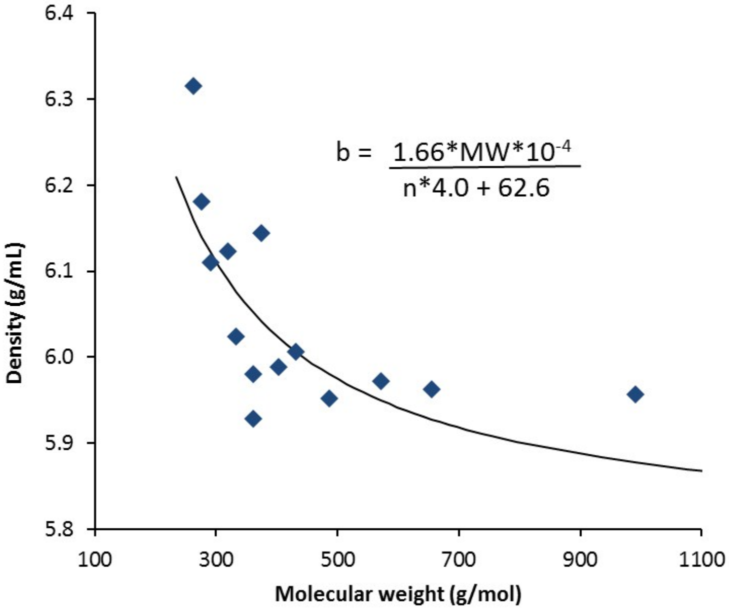

Fig. 2S. Density parameter b versus cation molecular weight for TDAC DCA ILs.

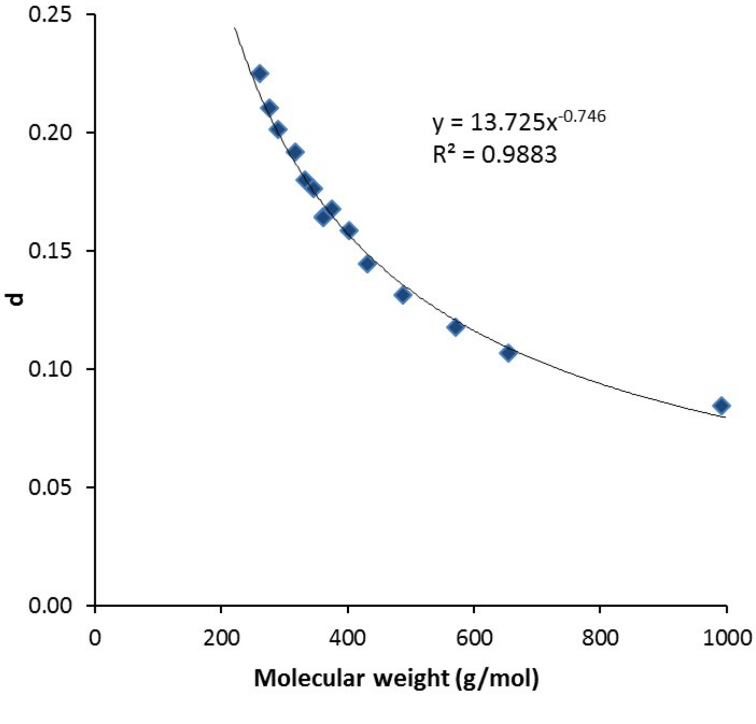

Fig. 3S. d (the intercept of ln(ρ) *versus* T plots) *versus* molecular weight for TDAC DCA ILs.

Table 4S. Viscosity data for TDAC DCA ILs.

Compound	Viscosity/cP							
	293 K	303 K	313 K	323 K	333 K	343 K	353 K	363 K
$[C_3(NEt_2)_3]DCA$ (**3b**)	64.2	41.9	28.3	19.9	14.6	11.4	8.9	7.1
$[C_3(NPr_2)_3]DCA$ (**3c**)	107[a]			21.4[a]	14.6	10.2	7.5	5.7
$[C_3(NBu_2)_3]DCA$ (**3d**)	293	160	95	60.6	41	29	21.5	16.4
$[C_3(NPent_2)_3]DCA$ (**3e**)	308	168	97.9	60.9	40	27	19.8	14.4
$[C_3(NHex_2)_3]DCA$ (**3f**)	332	183	109	69.4	47	32.9	23.5	17.8
$[C_3(NDec_2)_3]DCA$ (**3g**)	554	291	163	97.5	62.3	41.6	28.9	20.8
$[C_3(NBuMe)_3]DCA$ (**4a**)	101	58.6	36.6	24.1	16.9	12.4	9.47	7.3
$[C_3(NMe_2)_2(NPrMe)]DCA$ (**8a**)	107.3	61.3	38.2	25.1	17.5	12.8	10.1	8.02
$[C_3(NMe_2)_2(NBuMe)]DCA$ (**8b**)	67.4	40.3	25.3	17.2	12.1	9.14	7.05	5.57
$[C_3(NEt_2)_2(NBuMe)]DCA$ (**9a**)	73.7	45.8	30.2	20.8	15.3	11.5	8.9	7.1
$[C_3(NEt_2)_2(NHexMe)]DCA$ (**9b**)	86.2	52.8	34.4	23.4	17	12.7	9.78	7.71
$[C_3(NEt_2)_2(NMe_2)]DCA$ (**10a**)	58.4	37.1	25.4	17.9	13.2	10	7.94	6.4
$[C_3(NEt_2)_2(NBu_2)]DCA$ (**10b**)	105	63.3	40.8	27.4	19.4	14.2	11	8.6
$[C_3(NEt_2)_2(NHex_2)]DCA$ (**10c**)	131	76.8	48.4	32	22.2	16	12	9.3

[a] Calculated based on 60–90 °C data.

Table 5S. Viscosity fit parameters for TDAC DCA ILs.

Compound	Parameter					
	η_0	B	T_0	D	A	E_a
$[C_3(NEt_2)_3]DCA$ (**3b**)	0.078	962	150	6.4	0.447	28.9
$[C_3(NPr_2)_3]DCA$ (**3c**)	0.044	898	178	5.0	0.149	31.8
$[C_3(NBu_2)_3]DCA$ (**3d**)	0.133	898	176	5.1	0.036	38.7
$[C_3(NPent_2)_3]DCA$ (**3e**)	0.029	1318	151	8.7	0.017	40.6
$[C_3(NHex_2)_3]DCA$ (**3f**)	0.075	1100	162	6.8	0.042	38.6
$[C_3(NDec_2)_3]DCA$ (**3g**)	0.024	1445	149	9.7	0.009	43.7
$[C_3(NBuMe)_3]DCA$ (**4a**)	0.079	863	173	5.0	0.057	35.0
$[C_3(NMe_2)_2(NPrMe)]DCA$ (**8a**)	0.132	731	184	4.0	0.051	35.4
$[C_3(NMe_2)_2(NBuMe)]DCA$ (**8b**)	0.089	766	178	4.3	0.073	33.4
$[C_3(NEt_2)_2(NBuMe)]DCA$ (**9a**)	0.096	856	164	5.2	0.231	30.8
$[C_3(NEt_2)_2(NHexMe)]DCA$ (**9b**)	0.091	882	164	5.4	0.179	31.8
$[C_3(NEt_2)_2(NMe_2)]DCA$ (**10a**)	0.097	848	161	5.3	0.386	29.0
$[C_3(NEt_2)_2(NBu_2)]DCA$ (**10b**)	0.070	980	159	6.2	0.127	33.2
$[C_3(NEt_2)_2(NHex_2)]DCA$ (**10c**)	0.053	1063	157	6.8	0.076	34.9

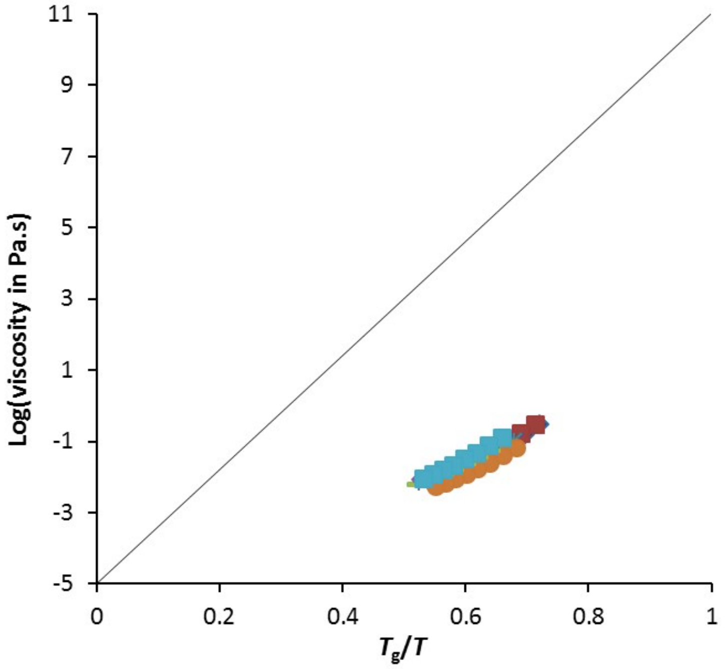

Fig. 4S. Fragility plot for TDAC DCA ILs.

Table 6S. Viscosities of non-TDAC cations with DCA.

Cation[a]	MW (g/mol)	Viscosity at 20 °C (cP)	Reference
C_1mim	163.2	18.6	1
C_2mim	177.2	19.1	1
C_4mim	205.3	36.7	1
C_6mim	233.3	59.9	1
1-Me-2-Et-Pyrazolium	177.2	29.8	1
Me_3S	143.2	27.2	2
$MeEt_2S$	171.3	22.9	2
$MePr_2S$	199.3	29.5	2
$MeBu_2S$	227.4	60.0	2
$EtMe_2S$	157.2	25.3	2
Et_3S	185.3	20.9	2
$EtPr_2S$	213.3	29.4	2
$EtBu_2S$	241.4	51.7	2
PY_{12}	180.3	30.3	1
PY_{13}	194.3	45	3
PY_{14}	208.3	50	3
PY_{16}	236.4	45	3

[a] mim = methylimidazolium; PY = pyrrolidinium

Table 7S. Conductivity data for TDAC DCA ILs.

Compound	Conductivity/mS.cm^{-1}							
	293 K	303 K	313 K	323 K	333 K	343 K	353 K	363 K
[$C_3(NEt_2)_3$]DCA (**3b**)	4.688	6.797	9.199	11.97	15.14	18.63	22.4	26.33
[$C_3(NPr_2)_3$]DCA (**3c**)					5.87	8.02	10.48	13.25
[$C_3(NBu_2)_3$]DCA (**3d**)	0.624	1.158	1.892	2.886	4.161	5.73	7.576	9.684
[$C_3(NPent_2)_3$]DCA (**3e**)	0.295	0.543	0.914	1.439	2.14	3.03	4.12	5.4
[$C_3(NHex_2)_3$]DCA (**3f**)	0.161	0.26	0.414	0.62	0.891	1.228	1.644	2.129
[$C_3(NDec_2)_3$]DCA (**3g**)	0.022	0.043	0.078	0.13	0.205	0.307	0.44	0.61
[$C_3(NBuMe)_3$]DCA (**4a**)	1.92	3.09	4.56	6.35	8.48	10.9	13.62	16.6
[$C_3(NMe_2)_2(NPrMe)$]DCA (**8a**)	3.17	5.42	8.44	12.38	16.9	22.5	28.3	35.0
[$C_3(NMe_2)_2(NBuMe)$]DCA (**8b**)	4.38	7.13	10.69	15.09	20.5	26.3	32.7	39.6
[$C_3(NEt_2)_2(NBuMe)$]DCA (**9a**)	3.501	5.119	7.134	9.503	12.2	15.24	18.6	22.16
[$C_3(NEt_2)_2(NHexMe)$]DCA (**9b**)	2.77	4.34	6.34	8.82	11.75	15.13	18.88	23.15
[$C_3(NEt_2)_2(NMe_2)$]DCA (**10a**)	4.643	6.969	9.537	12.44	15.7	19.25	23.13	27.21
[$C_3(NEt_2)_2(NBu_2)$]DCA (**10b**)	2.203	3.424	4.912	6.703	8.794	11.16	13.82	16.71
[$C_3(NEt_2)_2(NHex_2)$]DCA (**10c**)	1.028	1.71	2.572	3.641	4.939	6.463	8.21	10.164

Table 8S. Conductivity fit data for TDAC DCA ILs.

Compound	Parameter							
	σ_∞	B	T_0	D	δ	A	E_a	δ
[$C_3(NEt_2)_3$]DCA (**3b**)	489	551	174	3.2	0.0266	21644	20204	0.424
[$C_3(NPr_2)_3$]DCA (**3c**)	376	514	210	2.4	0.0034	94644	26776	0.100
[$C_3(NBu_2)_3$]DCA (**3d**)	605	730	187	3.9	0.0079	186853	29741	0.156
[$C_3(NPent_2)_3$]DCA (**3e**)	668	895	177	5.0	0.0048	267153	32583	0.086
[$C_3(NHex_2)_3$]DCA (**3f**)	489	1175	147	8.0	0.0033	51825	30452	0.025
[$C_3(NDec_2)_3$]DCA (**3g**)	419	1353	156	8.7	0.0009	271183	39112	0.005
[$C_3(NBuMe)_3$]DCA (**4a**)	441	580	186	3.1	0.0108	56048	24467	0.307
[$C_3(NMe_2)_2(NPrMe)$]DCA (**8a**)	904	535	198	2.7	0.0717	234232	26522	0.775
[$C_3(NMe_2)_2(NBuMe)$]DCA (**8b**)	808	499	198	2.5	0.0771	141550	24623	0.900
[$C_3(NEt_2)_2(NBuMe)$]DCA (**9a**)	556	619	171	3.6	0.0107	29307	21642	0.348
[$C_3(NEt_2)_2(NHexMe)$]DCA (**9b**)	742	642	178	3.6	0.0082	112681	25476	0.298
[$C_3(NEt_2)_2(NMe_2)$]DCA (**10a**)	382	464	188	2.5	0.0580	22792	20258	0.501
[$C_3(NEt_2)_2(NBu_2)$]DCA (**10b**)	452	608	179	3.4	0.0080	38131	23288	0.283
[$C_3(NEt_2)_2(NHex_2)$]DCA (**10c**)	459	718	175	4.1	0.0008	55828	25942	0.172

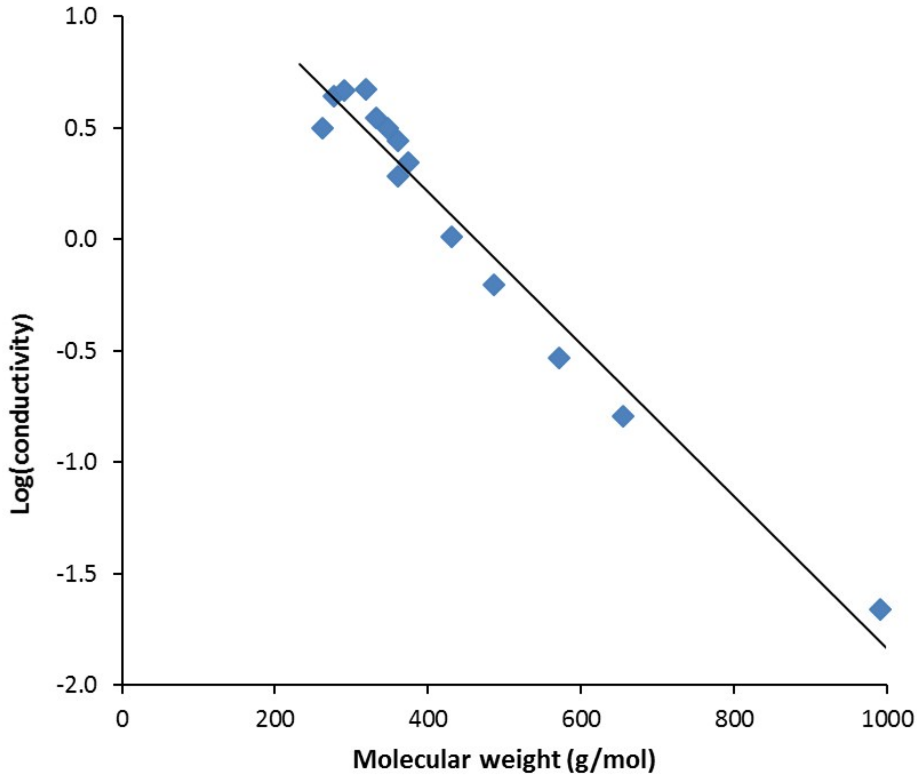

Fig. 5S. Plot of Log(conductivity) versus MW with a linear fit.

Table 9S. Walden parameters for TDAC DCA ILs.

Compound	Average ΔW	Slope
$[C_3(NEt_2)_3]$DCA (**3b**)	0.12	0.799
$[C_3(NPr_2)_3]$DCA (**3c**)	0.46	0.886
$[C_3(NBu_2)_3]$DCA (**3d**)	0.04	0.950
$[C_3(NPent_2)_3]$DCA (**3e**)	0.27	0.964
$[C_3(NHex_2)_3]$DCA (**3f**)	0.50	0.905
$[C_3(NDec_2)_3]$DCA (**3g**)	0.84	1.027
$[C_3(NBuMe)_3]$DCA (**4a**)	0.25	0.835
$[C_3(NMe_2)_2(NPrMe)]$DCA (**8a**)	0.10	0.937
$[C_3(NMe_2)_2(NBuMe)]$DCA (**8b**)	0.16	0.899
$[C_3(NEt_2)_2(NBuMe)]$DCA (**9a**)	0.18	0.807
$[C_3(NEt_2)_2(NHexMe)]$DCA (**9b**)	0.11	0.898
$[C_3(NEt_2)_2(NMe_2)]$DCA (**10a**)	0.20	0.810
$[C_3(NEt_2)_2(NBu_2)]$DCA (**10b**)	0.15	0.822
$[C_3(NEt_2)_2(NHex_2)]$DCA (**10c**)	0.28	0.875

X-ray crystallography

Single crystals of **3a** and **3c** formed in the neat liquid. A suitable crystal of each was selected and mounted on a SuperNova, Dual, Cu at zero, Atlas diffractometer. Using Olex2,[4] the structure was solved with the XS structure solution program[5] using Direct Methods and refined with the XL refinement package[5] using Least Squares minimisation.

Table 10S. Crystallographic data for **3a**.H_2O and **3c**.

	**3a**.H_2O	**3c**
Empirical formula	$C_{11}H_{20}N_6O$	$C_{23}H_{42}N_6$
Formula weight	252.33	402.63
Temperature (K)	120.01(10)	296(2)
Crystal system	Monoclinic	Monoclinic
Space group	*C*2/*c*	*C*2/*c*
a (Å)	23.2120(4)	21.8840(7) Å
b (Å)	9.00560(10)	9.4228(3) Å
c (Å)	13.5409(2)	27.0076(11) Å
α (°)	90	90°
β (°)	104.386(2)	113.472(2)°
γ (°)	90	90°
Volume (Å^3)	2741.81(7)	5108.4(3)
Z	8	8
Density (calculated) (mg mm^{-3})	1.223	1.047
Absorption coefficient (mm^{-1})	0.684	0.064
F(000)	1088	1776
Crystal size (mm^3)	0.307 × 0.27 × 0.098	0.51 × 0.50 × 0.38
Theta range for data collection	3.932 to 76.824°	3.3 to 55.0°
Index ranges	$-29 \le h \le 29$	$-28 \le \mathrm{h} \le 28$
	$-11 \le k \le 11$	$-12 \le \mathrm{k} \le 12$
	$-16 \le l \le 17$	$-35 \le \mathrm{l} \le 35$
Reflections collected	29797	55815
Independent reflections	2881 [*R*(int) = 0.0292]	5876 [R(int) = 0.0447]
Data / restraints / parameters	2881 / 2 / 175	5876 / 0 / 297
Goodness-of-fit on F^2	1.078	1.160
Final R indexes [$I \ge 2\sigma(I)$]	R_1 = 0.0373, wR_2 = 0.1079	R_1 = 0.0444, wR_2 = 0.1369
Final R indexes (all data)	R_1 = 0.0398, wR_2 = 0.1103	R_1 = 0.0744, wR_2 = 0.1665
Largest diff. peak/hole (e Å^{-3})	0.293/–0.365	0.340, –0.475

Table 11S. Bond lengths (Å) and angles (°) for $[C_3(NMe_2)_3]DCA.H_2O$ (**3a**.H_2O).

C1–C2	1.3801(13)	N1–C1	1.3274(12)
C1–C3	1.3833(13)	N2–C2	1.3305(12)
C2–C3	1.3813(13)	N3–C3	1.3235(13)
N1–C4	1.4566(12)	N1–C5	1.4595(12)
N2–C6	1.4562(13)	N2–C7	1.4576(12)
N3–C8	1.4544(12)	N3–C9	1.4552(12)
N5–C10	1.3073(14)	N4–C10	1.1580(14)
N5–C11	1.3065(14)	N6–C11	1.1583(14)
O1–H1A	0.862(13)	H1A---N4	2.027(14)
O1–H1B	0.868(13)	H1B---N6	2.092(14)
C2-C1-C3	59.98(6)	C1-N1-C5	119.34(8)
N1-C1-C2	148.97(9)	C1-N1-C4	121.20(8)
N1-C1-C3	151.00(9)	C4-N1-C5	118.22(7)
C1-C2-C3	60.12(6)	C2-N2-C6	119.12(8)
N2-C2-C1	148.68(9)	C2-N2-C7	120.93(8)
N2-C2-C3	151.04(9)	C6-N2-C7	118.74(8)
C2-C3-C1	59.89(7)	C3-N3-C9	122.09(8)
N3-C3-C1	150.30(9)	C3-N3-C8	121.13(8)
N3-C3-C2	149.80(9)	C8-N3-C9	116.77(8)
C11-N5-C10	121.41(9)	N6-C11-N5	173.45(11)
H1A-O1-H1B	106.2(15)	N4-C10-N5	173.31(10)
O1-H1A-N4	173.8(2)	O1-H1B-N6	168.8(2)

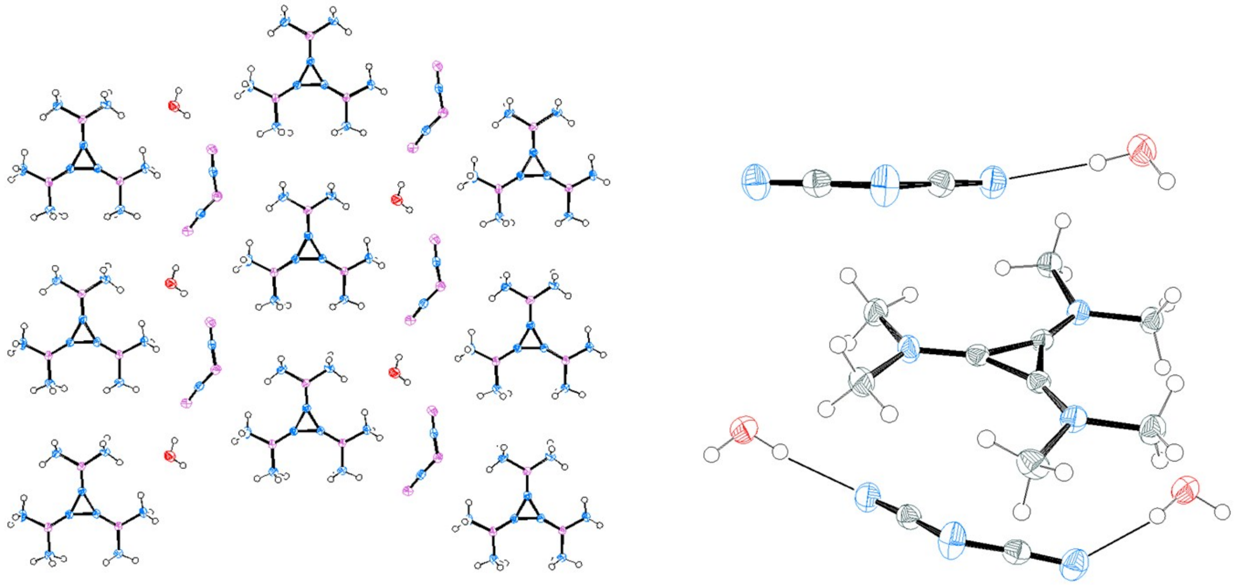

Fig. 6S. Illustration of one layer of **3a**.H_2O and the cation environment.

Table 12S. Bond lengths for $[C_3(NPr_2)_3]DCA$ (**3c**).

Bond		Length/Å	Bond		Length/Å
C1	C2	1.3801(18)	N1	C1	1.3301(17)
C1	C3	1.3738(19)	N2	C2	1.3286(17)
C2	C3	1.3752(19)	N3	C3	1.3266(18)
N1	C11a	1.4703(17)	N1	C12a	1.4649(17)
N2	C21a	1.4669(18)	N2	C22a	1.4635(18)
N3	C31a	1.4760(18)	N3	C32a	1.538(6)
			N3	C32a-	1.496(3)
C11b	C11a	1.5188(18)	C31b	C31a	1.520(2)
C11b	C11c	1.5187(19)	C31b	C31c	1.519(2)
C12a	C12b	1.5194(18)	C32a	C32b	1.508(9)
C12b	C12c	1.512(2)	C32b	C32c	1.551(13)
C21a	C21b	1.514(2)	C32a-	C32b-	1.515(3)
C21b	C21c	1.517(3)	C32b-	C32c-	1.508(5)
C22a	C22b	1.511(2)	C22b	C22c	1.528(2)
N4	C4	1.305(2)	C5	N4	1.311(2)
C4	N5	1.143(2)	C5	N6	1.1546(19)

Table 13S. Bond angles for $[C_3(NPr_2)_3]DCA$ (**3c**).

C3	C1	C2	59.92(10)	N2	C2	C3	149.86(13)
C3	C2	C1	59.81(10)	N2	C2	C1	150.25(13)
C1	C3	C2	60.27(9)	N3	C3	C1	150.02(14)
N1	C1	C3	150.07(13)	N3	C3	C2	149.69(13)
N1	C1	C2	149.92(13)	C2	N2	C22a	120.93(12)
C1	N1	C12a	120.51(11)	C2	N2	C21a	120.75(11)
C1	N1	C11a	120.57(11)	C22a	N2	C21a	117.93(11)
C12a	N1	C11a	118.35(10)	C3	N3	C32a-	118.39(13)
C3	N3	C31a	119.51(12)	C31a	N3	C32a	113.7(2)
C3	N3	C32a	123.0(2)	C31a	N3	C32a-	118.80(12)
N1	C11a	C11b	113.25(11)	C22a	C22b	C22c	111.32(15)
C11c	C11b	C11a	111.85(11)	N3	C31a	C31b	112.50(12)
N1	C12a	C12b	113.59(11)	C31c	C31b	C31a	111.11(12)
C12c	C12b	C12a	111.96(12)	C32b	C32a	N3	102.8(4)
N2	C21a	C21b	112.80(13)	C32a	C32b	C32c	111.0(6)
C21a	C21b	C21c	111.05(15)	N3	C32a-	C32b-	110.60(19)
N2	C22a	C22b	112.57(12)	C32c-	C32b-	C32a-	110.7(2)
N5	C4	N4	174.36(19)	C4	N4	C5	119.16(13)
N6	C5	N4	173.29(16)				

References

1. Yukihiro Yoshida, Osamu Baba, and Gunzi Saito, *J. Phys. Chem. B,* **2007**, 111*,* 4742–4749.
2. Dirk Gerhard, Samim Cenk Alpaslan, Heiner Jakob Gores, Marc Uerdingenc and Peter Wasserscheid, *Chem. Commun.*, **2005**, 5080–5082.
3. D. R. MacFarlane, S. A. Forsyth, J. Golding and G. B. Deacon, *Green Chemistry*, **2002**, 4, 444–448.
4. O. V. Dolomanov, L. J. Bourhis, R. J. Gildea, J. A. K. Howard and H. Puschmann, *J. Appl. Cryst*., 2009, **42**, 339.
5. G.M. Sheldrick, *Acta Cryst*., 2008, **A64**, 112.
